# Unveiling Racial Disparities in Localized Prostate Cancer: A Systems-Level Exploration of the lncRNA Landscape

**DOI:** 10.3390/genes16020229

**Published:** 2025-02-17

**Authors:** Rebecca A. Morgan, E. Starr Hazard, Stephen J. Savage, Chanita Hughes Halbert, Sebastiano Gattoni-Celli, Gary Hardiman

**Affiliations:** 1 Faculty of Medicine, Health and Life Sciences, School of Biological Sciences, Institute for Global Food Security (IGFS), Queen’s University Belfast (QUB), Belfast BT9 5DL, UK; rmorgan21@qub.ac.uk; 2 Academic Affairs Faculty, Medical University of South Carolina (MUSC), Charleston, SC 29425, USA; hazardes3@gmail.com; 3 Department of Urology, Medical University of South Carolina (MUSC), Charleston, SC 29425, USA; savages@musc.edu; 4 Ralph H. Johnson VA Health Care System (VAHCS) Medical Center, Charleston, SC 29425, USA; sebastiano.gattoni-celli@bms.com; 5 Department of Population and Public Health Sciences, University of Southern California, Los Angeles, CA 90033, USA; hughesha@usc.edu; 6 Norris Comprehensive Cancer Center, University of Southern California, Los Angeles, CA 90033, USA; 7 Department of Radiation Oncology, Medical University of South Carolina (MUSC), Charleston, SC 29425, USA; 8 Department of Medicine, Medical University of South Carolina (MUSC), Charleston, SC 29425, USA

**Keywords:** prostate cancer, racial disparities, long non-coding RNAs (lncRNAs), immune response, inflammation, differential gene expression, vitamin D supplementation, gene regulatory networks, biomarkers

## Abstract

Background/Objectives: Prostate cancer (PC) is the most common non-cutaneous cancer in men globally, and one which displays significant racial disparities. Men of African descent (AF) are more likely to develop PC and face higher mortality compared to men of European descent (EU). The biological mechanisms underlying these differences remain unclear. Long non-coding RNAs (lncRNAs), recognized as key regulators of gene expression and immune processes, have emerged as potential contributors to these disparities. This study aimed to investigate the regulatory role of lncRNAs in localized PC in AF men relative to those of EU and assess their involvement in immune response and inflammation. Methods: A systems biology approach was employed to analyze differentially expressed (DE) lncRNAs and their roles in prostate cancer (PC). Immune-related pathways were investigated through over-representation analysis of lncRNA–mRNA networks. The study also examined the effects of vitamin D supplementation on lncRNA expression in African descent (AF) PC patients, highlighting their potential regulatory roles in immune response and inflammation. Results: Key lncRNAs specific to AF men were identified, with several being implicated for immune response and inflammatory processes. Notably, 10 out of the top 11 ranked lncRNAs demonstrated strong interactions with immune-related genes. Pathway analysis revealed their regulatory influence on antigen processing and presentation, chemokine signaling, and ribosome pathways, suggesting their critical roles in immune regulation. Conclusions: These findings highlight the pivotal role of lncRNAs in PC racial disparities, particularly through immune modulation. The identified lncRNAs may serve as potential biomarkers or therapeutic targets to address racial disparities in PC outcomes.

## 1. Introduction

In 2020, there were an estimated 1,414,259 new cases of prostate cancer (PC) and 375,304 related deaths worldwide [1]. The disease remains a global health problem, however, persistent racial differences in disease presentation pose a significant challenge. AF men have increased risk of developing PC and suffer a greater mortality rate than EU men. In the USA, it is estimated that black males will have a 1 in 25 chance of dying from PC compared to 1 in 45 for non-Hispanic white males [2]. In the UK, racial disparities also persist where men of African descent (AF) are 2–3 times more likely to develop PC in comparison to Caucasian men as well as there being a 30% higher mortality rate [3]. Despite these differences, the underlying causes of this disparity remain unclear, and prostate cancer racial statistics have shown little change over the past two decades [4]. The limited availability of genomic data from AF patients compared to those of European ancestry (EU) poses a significant challenge for PC research. This problem is compounded by a long-standing history of medical distrust in black communities, particularly in the USA [5]. A striking example of this disparity in cancer data is highlighted by Spratt et al., who analyzed 5729 samples of common cancers from The Cancer Genome Atlas (TCGA) to assess the ethnic diversity of patients. Overall, only 12% (n = 660) of samples available were categorized as being from black patients in comparison to 77% (4389) from white patients [6].

Long non-coding RNAs (lncRNAs) are today recognized as potential biomarkers for multiple cancer types, which is a far cry from their initial classification as junk transcriptional noise lacking biological relevance. In the past, lncRNAs were simply categorized as transcripts longer than 200 nucleotides and were defined as not playing any role in transcriptional processes. It is only in recent years, with the realization that many play a fundamental role in cancer progression, that lncRNA functions and specific roles have emerged [7,8,9,10,11].

Network biology has become a vital technique to decipher complex biological systems [12,13,14,15,16]. The recognition that lncRNAs are key regulators of multiple mRNA targets has made network biology an ideal approach for analysis and visualization of lncRNA interactions. Centrality measures the degree of a node in a network, defined as the number of edges it is connected to. In simple terms, it quantifies a node’s importance or influence within the network based on its connections. To determine centrality, numerical values are assigned to each node and edge. Nodes with the highest degree, meaning the greatest number of connections, are considered the most central in the network. In the context of ncRNA–mRNA interaction networks, centrality metrics, such as in-degrees and out-degrees, highlight key players that exhibit extensive interactions with other nodes. These influential elements often play pivotal roles in biological processes or disease-specific pathways [17,18]. Another critical metric in network analysis is structural equivalence, which assesses the similarity between nodes based on their connectivity. Two nodes are considered structurally equivalent if they share identical or highly similar relationships with the same set of nodes. Structural equivalence is, thus, particularly useful for identifying clusters or collaborative groups within a network [19]. This is a widely used algorithm within social media and online dating sites to match users based on their likes and dislikes [17,20]. The metric of structural equivalence allows identification of lncRNAs that collaborate to regulate target mRNAs [19,21], and, additionally, the ranking of lncRNAs based on their similarity of target mRNA regulation [15]. In lncRNA–mRNA interaction networks, this can reveal groups of lncRNAs that share common mRNA targets, providing insights into their cooperative functions [15].

Vitamin D deficiency is considered a risk factor for PC [22,23,24,25,26,27,28]. Individuals of African descent are significantly more prone to severe vitamin D deficiency than individuals of European descent. Studies have shown that AF individuals can have as much as a 15 to 20 times greater chance of suffering from severe vitamin D deficiency in comparison to those of EU [23]. This disparity is caused by two factors: the lack of a nutritionally rich vitamin D diet and increased skin pigmentation [29]. Darker skin tones have higher levels of melanin present, a pigment that absorbs and scatters ultraviolet-B (UB-V) radiation from the skin. Thus, it is more challenging for individuals with darker skin tones to produce vitamin D. Vitamin D deficiency is defined as being when serum levels of 25(OH)D are at <50 nmol (<20 ng/mL). Consequently, most AF men are vitamin D deficient [30].

Previously, we examined racial differences in the transcriptome of localized PC patients using a systems biology approach. This led to the discovery that AF men had higher gene expression of genes involved in immune response and inflammation, suggesting that these processes were contributing to the more severe disease progression observed in the AF men [24]. In this study, we examined the same patient cohort to investigate the regulatory role of lncRNAs, with a focus on racial differences and vitamin D deficiency. Using a systems biology and network-based approach, we sought to identify key lncRNAs that exert the most significant influence on mRNA expression in AF with localized prostate cancer. Additionally, we analyzed the lncRNAs modulated by vitamin D supplementation in AF and their role in regulating gene expression.

## 2. Materials and Methods

### 2.1. Patients and Sample Preparation

The patient samples used in this study were obtained from an earlier study that examined the effects of vitamin D supplementation on mRNA expression between AF and EU PC patients [10]. This study enrolled male participants diagnosed with localized prostate cancer and comprised 27 subjects (10 African American and 17 European American men) who had opted for prostatectomy as definitive treatment. As per standard care guidelines, a two-month interval between biopsy and prostatectomy was observed to allow resolution of biopsy-induced inflammation. Participants were randomized to receive either 4000 IU per day of vitamin D3 (Carlson Laboratories, Lincolnshire, IL, USA) or a placebo for two months prior to surgery. This dosage is supported by our previous research investigating the effects of vitamin D supplementation on various health outcomes and particularly in addressing hypovitaminosis D, a prevalent health disparity among AF [31].

Blood samples were collected from each participant at enrollment and on the day of surgery to measure serum levels of 25-hydroxyvitamin D3 (25[OH]D3) in nanograms per milliliter (ng/mL). Of the total participants, 14 (five African American and nine European American men) received vitamin D3 supplementation, while 13 (five African American and eight European American men) received a placebo. Exit serum levels of 25(OH)D confirmed high compliance among all enrolled subjects. This study was approved by the Institutional Review Board (IRB) of the Medical University of South Carolina, USA (MUSC; SC, USA), by the Ralph H Johnson VA Medical Center (VAMC; SC, USA), and by the Research and Development (R&D) Committee of the VAMC. This interventional study was performed under investigational new drug (IND) 77839 terms, as set out by the US FDA. Prostate tissue samples from these patients were collected, and RNA extracted and subjected to paired end short read sequencing [10]. Figure S1 provides an overview of how the RNA-seq data were generated. Supplemental Table S1 provides details on the clinical characteristics of the patients in this study.

### 2.2. RNA-seq Preparation and Differential Expression Analyses

Raw fastq files underwent pre-processing using the following pipeline. FastQC (version 0.11.8) was used to provide a quality report on the samples [32]. Cutadapt (version 1.18) was utilized to remove low-quality nucleotide and adapter sequences identified from FastQC [33]. Both paired- and single-end reads were processed by Cutadapt using consistent quality control measures. Patient samples were then aligned to the ENSEMBL Homo sapiens genome (GRCh38.p13) using the STAR RNA-Seq aligner (version 2.5.3a) and sorted by coordinate/position [34]. HTSeq-count was used to (version 0.11.1) generate count data [35]. The R environment (version 1.2.1335) and the DESeq2 package (version 3.6) were used to test for differential expression (DE) [36,37].

In total, three separate DE analyses were performed: 1. AF vs. EU with the AF set as the test group, 2. AF vitamin D supplemented vs. AF placebo with AF vitamin D supplemented set as the test group, and, lastly, 3. EU vitamin D supplemented vs. EU placebo with EU vitamin D supplemented set as the test group. The Gene Ontology tool Biomart was used to annotate the DE results with HUGO symbols and biotype classifications [38]. lncRNAs were filtered based on ENSEMBL biotype classification lists [39]. With annotated DE transcripts, a significance threshold (q ≤ 0.1 and a linear fold change of ≥1.5) was set to generate significantly DE transcripts in the AF vs. EU analysis. A q value threshold of ≤0.4 and a linear fold change value of ≥1.5 were used in each vitamin D vs. placebo analysis. Supplemental Figure S2 is a schematic of the RNA-seq pre-processing and DESeq analyses completed.

### 2.3. Network Analysis Using Centrality Metrics

The lncRNAs with the greatest impact on target mRNAs were identified using data from the TCGA Prostate Adenocarcinoma (PRAD)long non-coding RNA Heterogeneous Regulatory Network integrator (LongHorn) algorithm [15,40]. LongHorn’s lncRNA–mRNA predictions are based on reverse-engineered canonical interactions identified through the Encyclopedia of DNA Elements (ENCODE) project [15,40].

Differentially expressed (DE) transcripts were filtered and categorized into lncRNAs and mRNAs, generating two intersections: one between LongHorn results and significantly DE lncRNAs, and another between LongHorn results and significantly DE mRNAs. These intersections were used to assess the influence of lncRNAs through network centrality measures, ranking them accordingly. Cytoscape (version 3.7.1) was then employed to visualize the effects of the top-ranked lncRNAs on their target mRNAs [41].

**Figure 1:**
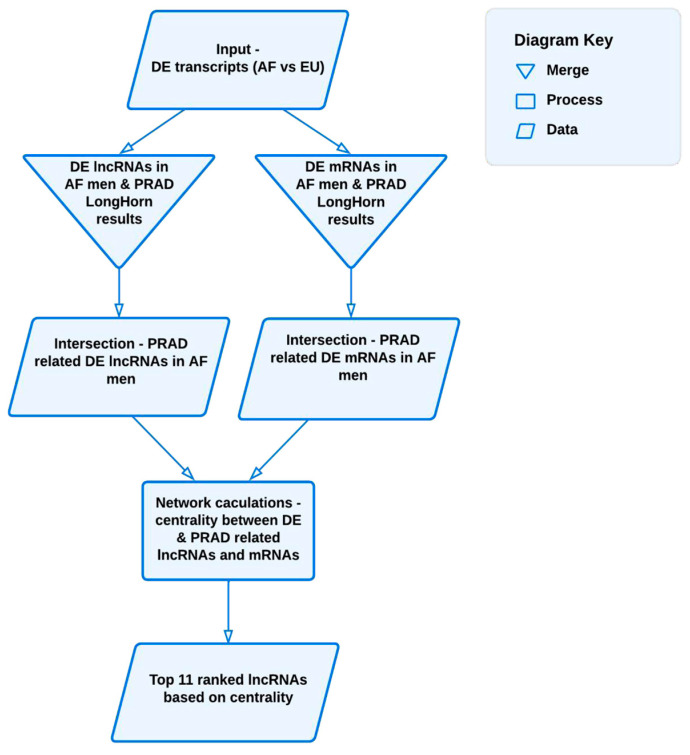
To identify key long non-coding RNAs (lncRNAs) influencing mRNA expression differences between African (AF) and European (EU) prostate cancer patients, we implemented a network centrality workflow. Differentially expressed (DE) transcripts were first identified using DESeq2 analysis. These DE lncRNAs and mRNAs were then intersected with predictions from the LongHorn PRAD lncRNA–mRNA algorithm. By calculating centrality metrics within this integrated network, we ranked lncRNAs based on their regulatory impact, highlighting those most influential in the observed expression disparities between AF and EU cohorts.

Figure 1 illustrates the workflow and results generated by the AF vs. EU DESeq2 analysis. The LncRNA Systems Biology Analyses are described in more detail in the Supplementary Materials [42,43,44,45,46,47,48,49,50,51,52,53,54]. Supplemental Table S2 outlines CbioPortal PC datasets queried. Figure S2B is a schematic of the workflow outlining downstream analyses of the top-ranking lncRNAs.

### 2.4. Systems-Level Analyses

Pathway impact analyses were performed using Advaita iPathwayGuide [53] and over-representation analyses using Toppfun [54]. Both tools were also used to complete functional enrichment analyses to identify biological processes, pathways, and Gene Ontology (GO) terms enriched by the DE lncRNA regulated target mRNAs from both comparisons (i.e., AF vs. EU and AF vitamin D supplemented vs. placebo). REVIGO was used to visualize the significant GO biological processes [55].

### 2.5. Gene Ontology Comparisons

Jvenn was used to create area-proportional Venn diagrams to identify common and unique DE lncRNAs between each of the DE analysis contexts. GO terms identified from all DE transcripts for AF men and AF men supplemented with Vitamin D from ToppFun were compared using jvenn [56].

To identify which lncRNAs regulate mRNAs enriching specific GO terms, we integrated genes associated with common GO terms with differentially expressed lncRNAs. This approach allowed us to pinpoint lncRNAs involved in the regulation of genes mapping for each GO term.

## 3. Results

### 3.1. lncRNA Differential Expression

**Figure 2:**
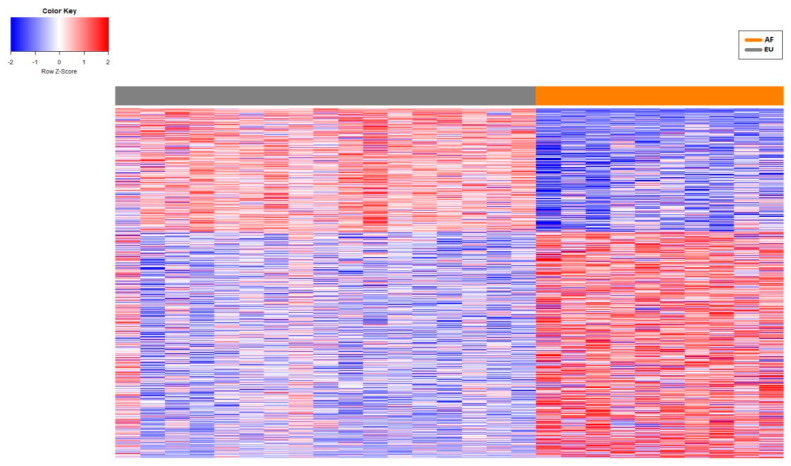
Differential expression of lncRNAs between patients of African (AF) and European descent (EU). A total of 1283 lncRNAs were significantly DE between AF and EU patients. Red and blue boxes indicate relative over- and under expression with respect to a reference that is calculated as the midpoint between the AF and EU groups. Only lncRNA transcripts were found to be significant at the level q ≤ 0.1 and with an absolute fold change ≥ 1.5 in the comparison, are shown.

To determine if lncRNA expression significantly differed between patients of AF and EU descent, a significance threshold (q ≤ 0.1 and fold change ≥ 1.5) was applied to the DESeq2 (AF vs. EU) results. A total of 5850 transcripts were identified as significantly DE (Supplemental Table S3), with 1283 being classified as lncRNAs (Figure 2 and Supplemental Table S4). Most of these DE lncRNAs were classified as antisense lncRNAs (48%) or lincRNAs (44%) (Supplemental Figure S3). The earlier study identified 3107 significantly DE transcripts using a q value of ≤0.1 between patients of AF and EU descent [10]. The contrast between these results is due to the different tools, genome build, and aligner employed. This study utilized the STAR aligner rather than TopHat2, which was used in the earlier study [57]. In contrast, this new study employed the more recent ENSEMBL GRCh38.

### 3.2. Network Analysis

With significantly differentially expressed (DE) lncRNAs identified in AF men, the next step was to determine their interactions with and modulation of mRNAs. This was achieved by merging the significantly DE lncRNAs and mRNAs identified in AF men with the lncRNA–mRNA interaction results from the TCGA PRAD algorithm. The intersection yielded a list of significantly DE lncRNAs and their predicted mRNA targets based on the LongHorn-PRAD algorithm. The analysis revealed that 142 DE lncRNAs in AF men exhibited 4158 interactions with mRNAs, including 153 uniquely upregulated and 55 uniquely downregulated mRNA targets (Supplemental Tables S5 and S6). The resulting 4158 mRNA targets were subject to over-representation analysis to aid biological interpretation. Upregulated genes revealed processes related to immune response and inflammation. REViGO was utilized to reduce biological redundancy by clustering the GO biological processes based on semantic similarity. This revealed the positive regulation of immune system process (GO: 0002684), regulation of cell activation (GO: 0050865), defense response (GO: 0006952), regulation of defense response (GO: 0031347), regulation of response to external stimulus (GO: 0032101), response to other organism (GO: 0051707), T-cell migration (GO: 0072678), response to biotic stimulus (GO: 0009607), and mononuclear cell proliferation (GO: 0032943), as shown in Figure S3, Table 1, and Supplemental Table S7. Over-representation of the downregulated mRNA targets did not result in enrichment of biological processes.

With mRNA targets identified for 142 DE lncRNAs in AF patients (Supplemental Table S5), we subsequently wanted to establish which of these lncRNAs were most important in terms of centrality, i.e., the number of edges connected to a node.

**Figure 3:**
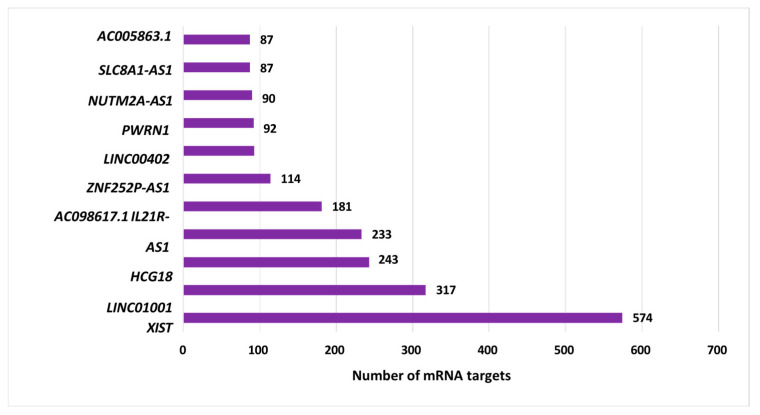
Top-ranking lncRNAs. The top 11 long non-coding RNAs (lncRNAs) were ranked based on their centrality. These interactions were constructed using the TCGA Prostate Adenocarcinoma (PRAD) dataset, applying the LongHorn algorithm, and incorporated DE lncRNAs and mRNAs identified in AF patients compared to their EU counterparts.

The top 11 ranking lncRNAs in terms of centrality (listed in descending order) were *XIST* (interacting with 574 mRNAs), *LINC01001* (317 mRNAs), *HCG18* (243 mRNAs), *IL21R-AS1* (233 mRNAs), *AC098617.1* (181 mRNAs), *ZNF252P-AS1* (114 mRNAs), *LINC00402* (93 mRNAs), *PWRN1* (92 mRNAs), *NUTM2A-AS1* (90 mRNAs), *SLC8A1-AS1* (87 mRNAs), and *AC005863.1* (87 mRNAs) (Supplemental Table S8). Figure 3 displays the top 11 lncRNAs ranked by centrality using the AF vs. EU results.

Chromosomal locations of the top-ranking lncRNAs were identified with *ZNF252P-AS1* and *NUTM2A-AS1*, with both being located at PC genomic hotspots. *ZNF252P-AS1* resides within the 8q24 region, which is widely recognized as a genomic locus for PC susceptibility, particularly in AF men with PC [58]. *NUTM2A-AS1* is located within the 10q23 region, which harbors the tumor suppressor gene *PTEN* [Phosphatase and Tensin Homolog]. Deletions or allelic losses containing *PTEN* occur in 20–30% of PC cases [59,60]. Loss of PTEN function leads to the suppression of the PI3K-Akt signaling pathway, which is closely associated with poor clinical outcomes in prostate cancer [59]. The chromosomal locations of the top-ranking lncRNAs are provided in Table 2.

**Figure 4:**
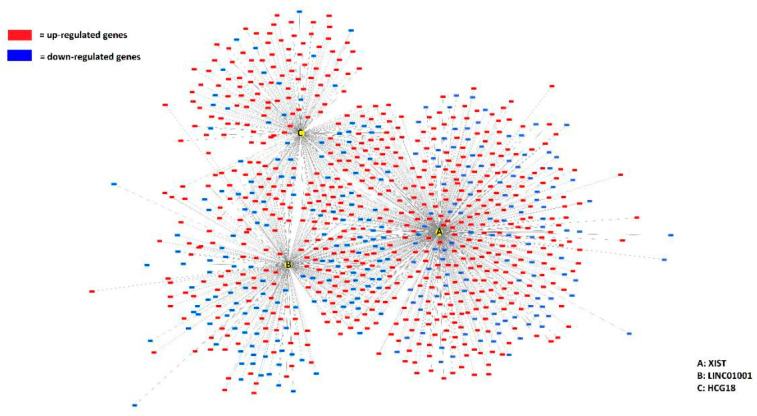
Top-three-ranking lncRNAs (*XIST*, *LINC01001, and HCG18*) interact with the greatest number of mRNA targets. Graphical representation of the top three lncRNAs (A: *XIST*, B: *LINC01001*, and C: *HCG18*) and their interaction with target mRNAs in AF men. Red: upregulated and Blue: downregulated.

Over-representation analyses revealed an association with immune response and inflammatory processes, prompting further investigation into whether the top-ranked lncRNAs in AF patients could be classified as immune response-related. Combining the top-ranking lncRNAs revealed that all 11 lncRNAs could be categorized as immune response-related (Supplemental Table S9). Further analysis revealed that the top three ranked lncRNAs—*XIST*, *LINC01001*, and *HCG18*—possess the ability to modulate gene expression (Figure 4).

Over-representation analysis using the mRNA targets of *XIST*, *LINC01001,* and *HCG18* revealed processes related to immune response including cell activation (GO: 0001775), leukocyte mediated immunity (GO:0002443), leukocyte activation (GO: GO:0045321), myeloid leukocyte activation (GO:0002274), and immune effector process (GO:0002252). The additional lncRNA–mRNA networks (*IL21R-AS1*, *AC098617.1*, *ZNF252P-AS1,* and *LINC00402*) are presented in Supplemental Figure S4 and *PWRN1*, *NUTM2A-AS1*, *SLC8A1-AS1,* and *AC005863.1* are presented in Supplemental Figure S5. Over-representation analysis using the mRNA targets of *IL21R-AS1*, *AC098617.1*, *ZNF252P-AS1,* and *LINC00402* revealed more processes related to immune response, including T-cell differentiation (GO:0030217), cytoplasmic translation (GO:0002181), leukocyte differentiation (GO:0002521), lymphocyte differentiation (GO:0030098), and regulation of leukocyte differentiation (GO:1802105). Over-representation analysis using the mRNA targets of *PWRN1*, *NUTM2A-AS1*, *SLC8A1-AS1,* and *AC005863.1* again revealed processes related to cell activation (GO: 0001775), leukocyte activation (GO: 00453121), regulation of immune system process (GO:0002682), regulation of cell activation (GO: 0050865), and lymphocyte activation (GO: 0046649). Significant (FDR corrected) immune related pathways were identified using Advaita iPathwayGuide. These included antigen processing and presentation q = 8.143^−7^, chemokine signaling pathway q = 6.290^−6^, and ribosome q = 7.754^−6^.

**Figure 5:**
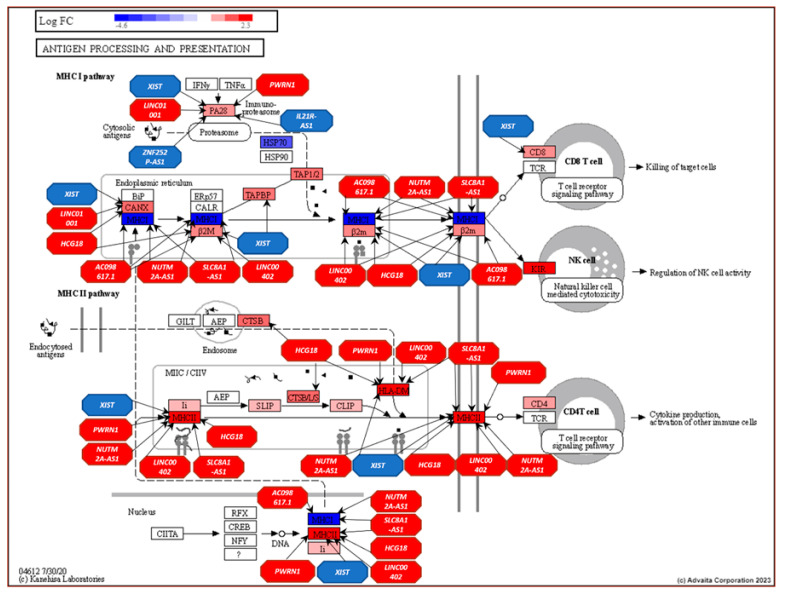
Antigen processing and presentation pathway (KEGG: 04612) displaying DE gene expression between AU and EU and the influence of the top-ranking lncRNAs in the prostate. Red: upregulated and Blue: downregulated. Genes are presented as rectangles and lncRNAs as octagons.

Within the antigen processing and presentation pathway, 10 of the top 11 lncRNAs—*XIST*, *LINC01001*, *HCG18*, *IL2IR-AS1*, *AC098617.1*, *ZNF252P-AS1*, *LINC00402*, *PWRN1*, *NUTM2A-AS1*, and *SLC8A1-AS1*—interacted with 12 genes, including *HLA-DOA*, *HLA-DPA1*, *HLA-DMA*, *CTSB*, *CANX*, *TAPBP*, *HLA-DPB1*, *HLA-F*, *B2M*, *PSME2*, *CD8A*, and *PSME1*. Figure 5 illustrates these interactions within the pathway.

**Figure 6:**
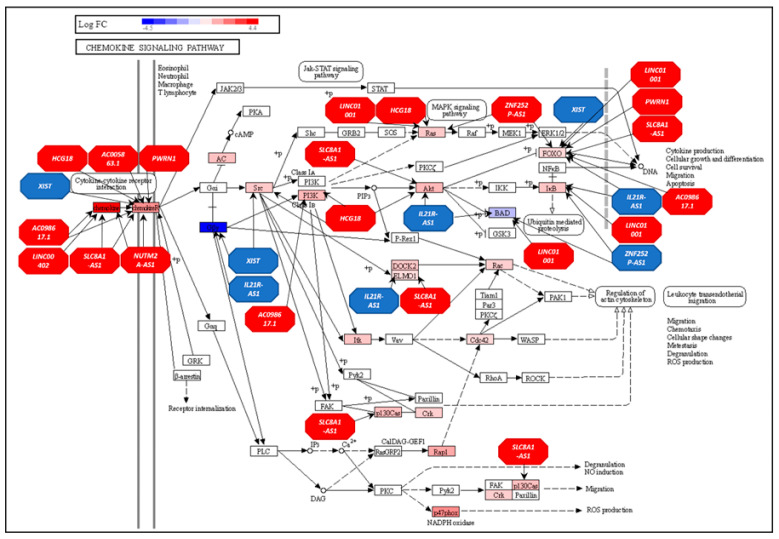
displays the chemokine signaling pathway with all top-ranking lncRNAs and their interaction with pathway genes. Genes are presented as rectangles and lncRNAs as octagons.

In the chemokine signaling pathway, (Figure 6) all 11 top-ranked lncRNAs interacted with 28 genes, such as *CCL17*, *CCL4*, *CCL5*, *NCF1*, *CCR4*, *CCR6*, *CCL22*, *GNG5*, *CCL3*, *XCR1*, *CCR5*, *CXCR4*, *BAD*, *AKT3*, *PIK3CG*, *CXCR6*, *CCR2*, *BCAR1*, *ADCY4*, *NFKBIB*, *HRAS*, *HCK*, *PIK3R5*, *CRK*, *RAC2*, *GNG2*, *FOXO3*, and *ELMO1*. These findings highlight the influential roles of the top-ranked lncRNAs in modulating immune-related pathways.

**Figure 7:**
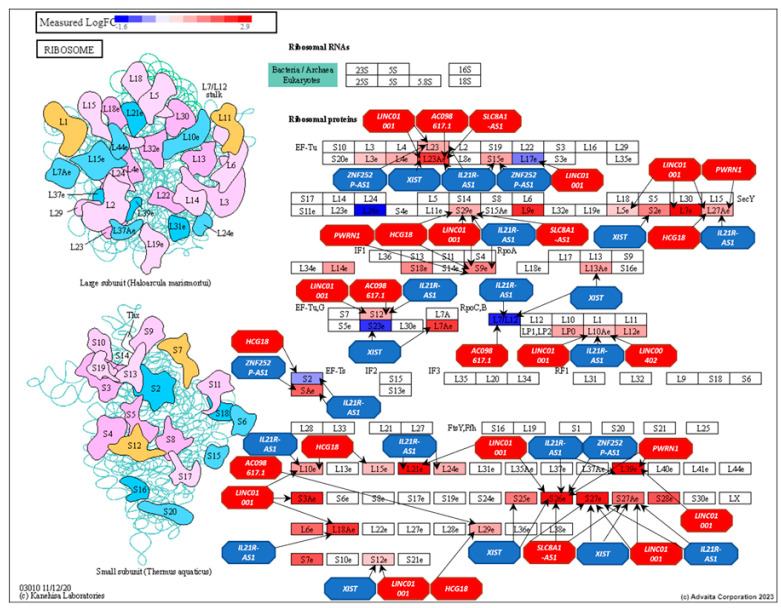
Ribosome pathway (KEGG: 03010) displaying DE gene expression between AF and EU prostate differential expression analysis and the influence of the top-ranking lncRNAs on their regulation. RED: upregulated and BLUE: downregulated. Genes are presented as rectangles and lncRNAs as octagons.

In the ribosome pathway (Figure 7), 9 of the top 11 ranked lncRNAs—*XIST*, *LINC01001*, *HCG18*, *IL2IR-AS1*, *AC098617.1*, *ZNF252P-AS1*, *LINC00402*, *PWRN1*, and *SLC8A1-AS1*—interacted with 31 genes.

These genes included *RPL39*, *RPL7*, *RPL21*, *RPS3A*, *RPL18A*, *RPL23A*, *RPL7A*, *RPSA*, *RPS27A*, *RPL26*, *RPS25*, *RPS2*, *RPS9*, *MRPL12*, *RPS23*, *RPL10*, *RPS15*, *RPL13A*, *RPL27A*, *RPL29*, *RPL26L1*, *MRPL23*, *MRPS12*, *RPS29*, *RPL24*, *RPL17*, *RPL15*, *RPL10A*, *MRPS2*, *RPS12*, and *RPL5.* These interactions highlight the significant influence of these lncRNAs within the ribosome pathway.

### 3.3. Analysis of the Top-Ranking lncRNAs

By comparing the top 11 ranked lncRNAs with findings from other studies investigating differential expression (DE) between AF and EU prostate cancer patients, we identified five lncRNAs—*AC098617.1*, *LINC00402*, *SLC8A1-AS1*, *AC005863.1*, and *LINC01001*—that were consistently DE across all datasets, as shown in Table 3.

To determine whether these lncRNAs could influence protein–RNA interactions, we employed CatRapid omics. CatRapid analysis predicted that 10 of the top 11 lncRNAs—*XIST*, *LINC01001*, *HCG18*, *IL21R-AS1*, *AC098617.1*, *LINC00402*, *PWRN1*, *NUTM2A-AS1*, *SLC8A1-AS1*, and *AC005863.1*—interacted with proteins. The results highlighted associations with immune and defence response-related proteins, while others predicted that lncRNA–protein interactions were linked to functions such as mRNA processing, RNA splicing, RNA exporting, and cell differentiation (Supplemental Table S10).

The top-ranked lncRNAs were analyzed against PC datasets hosted on the cBioPortal platform, excluding *AC098617.1* and *AC005863.1* as they were not recognized transcripts within cBioPortal [63]. The analysis revealed co-occurrence among several lncRNAs, as summarized in Table 4. The alteration frequency of these lncRNAs across these prostate cancer datasets, highlighting alterations in expression, structural variant, mutation, and copy number alteration (CAN), are provided in Supplemental Figures S9–S17.

Using structural equivalence analysis of these 11 lcRNAs, we identified that the four most impactful lncRNAs—*XIST*, *LINC01001*, *HCG18*, and *IL21R-AS1*—shared a high degree of similarity based on their shared mRNA targets. This indicates potential collaborative relationships among these lncRNAs in terms of regulating these targets.

**Figure 8:**
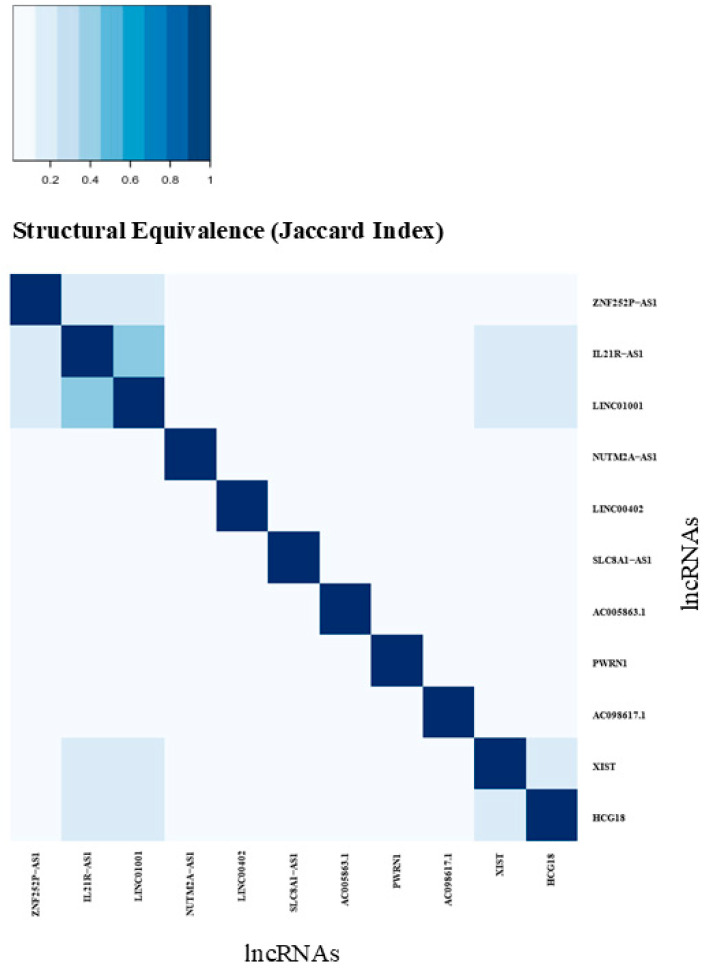
Structural equivalence of the top 11 ranking lncRNAs. Similarity plot of lncRNAs clustered by mRNA target similarity. Darker colors represent higher similarity among the target mRNAs.

Additionally, the analysis revealed that *LINC01001* and *IL21R-AS1* exhibited the highest similarity among the top 11 centrally ranked lncRNAs, suggesting a particularly strong cooperative relationship between these two. Figure 8 presents a similarity plot clustering the top 11 lncRNAs based on structural equivalence, while Figure S6 displays a dendrogram illustrating their relationships according to shared mRNA targets.

### 3.4. Structural Equivalence (AF vs. EU)

Using the structural equivalence metric, several lncRNAs DE between AF and EU were identified that share similar relationships with other nodes in the network but were not among the top 11 ranked lncRNAs identified through the centrality metric. This highlights the importance of analyzing relational patterns to uncover functionally relevant lncRNAs that may not be prominent based on centrality alone.

These newly identified lncRNAs include *AC104024.1*, *AC094125.4*, *LINC00877*, *DNM3OS*, *LINC00539*, *ATP1B3-AS1*, *FGF13-AS1*, *AC107079.1*, *GK-AS1*, *COL4A2-AS1*, *FRMD6-AS2*, *HIF1A-AS2*, *AP001627.1*, *LINC00882*, *LINC00987*, *ATXN8OS*, *AC090587.2*, *PCA3*, *PCCA-AS1*, *RAI1-AS1*, *LINC01068*, *LINC00887*, *HILCS-IT1*, *DDX11-AS1*, *AC144831.1*, *LINC00299*, *LINC00115*, *AP000439.2*, *FAM66C*, *HPN-AS1*, *LINC00313*, *LUCAT1*, *LINC00926*, *ZBTB20-AS4*, *LINC00494*, *CAMTA1-IT1*, *MIR497HG*, *LI-PE-AS1*, *FLG-AS1*, *SLC8A1-AS1*, *SNAP25-AS1*, *F11-AS1*, and *INTS6-AS1.* Figure S7 displays a similarity plot of the lncRNAs clustered by their structural equivalence to mRNA targets. Figure S8 provides a dendrogram of the structural equivalence of the lncRNAs based on their number of shared mRNA targets.

Over-representation analysis of the mRNA targets of these lncRNAs highlighted their involvement in processes related to immune response and inflammation. The identified processes included leukocyte activation (GO:0045321), T-cell activation (GO:0042110), leukocyte-mediated immunity (GO:0002443), T-cell differentiation (GO:0030217), lymphocyte activation (GO:0046649), immune effector process (GO:0002252), positive regulation of leukocyte activation (GO:0002696), regulation of leukocyte activation (GO:0002694), positive regulation of immune system processes (GO:0002684), and leukocyte activation involved in immune response (GO:0002366).

Among these lncRNAs, *AC084125.4* was located at 8q24.3, a well-known chromosomal hotspot for prostate cancer. The chromosomal distribution of the identified lncRNAs varied, with chromosome 13 harboring the largest number. The detailed chromosomal locations of lncRNAs identified through the structural equivalence metric are listed in Table 5.

Building on the over-representation analysis of the mRNA targets of these lncRNAs, which were associated with immune response processes, we next sought to determine whether they exhibited similar influence on the antigen processing and presentation, chemokine signaling, and ribosome pathways as the top-ranked lncRNAs identified in our study.

By combining the mRNA targets of these lncRNAs with the significant DE genes in these immune-related pathways, we identified several structurally equivalent lncRNAs that played a role in gene regulation.

Within the antigen processing and presentation pathway, 5 out of the 43 structurally equivalent lncRNAs—*LINC00115*, *F11-AS1*, *LIPE-AS1*, *SNAP25-AS1*, and *INTS6-AS1*—were found to interact with 6 genes: *HLA-DMA*, *CTSB*, *HLA-E*, *HLA-F*, *B2M*, and *PSME2*.

**Figure 9:**
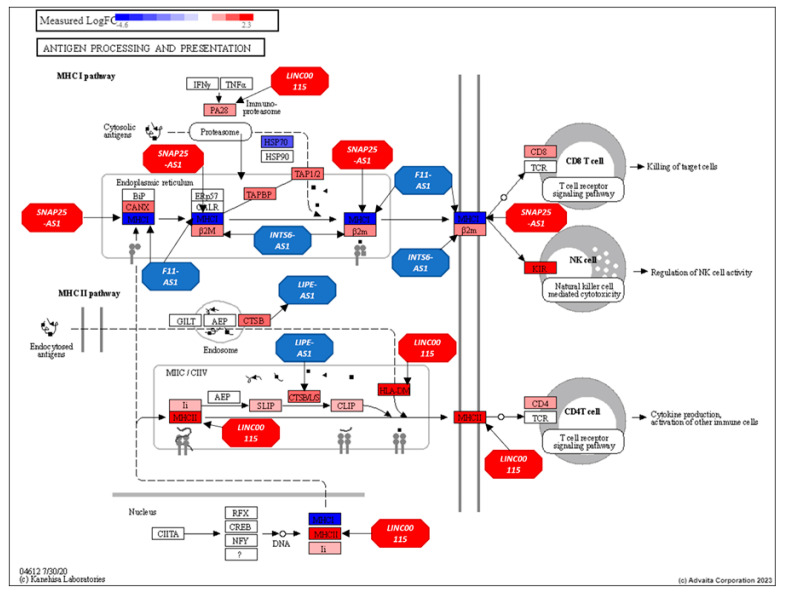
Antigen processing and presentation pathway (KEGG: 04612) displaying gene regulation of AF vs. EU prostate differential expression analysis and the influence of lncRNAs identified using structural equivalence analyses. Red: upregulated and Blue: downregulated. Genes are presented as rectangles and lncRNAs as octagons.

Figure 9 illustrates the antigen processing and presentation pathway, highlighting these structurally equivalent lncRNAs and their interactions with the pathway genes.

**Figure 10:**
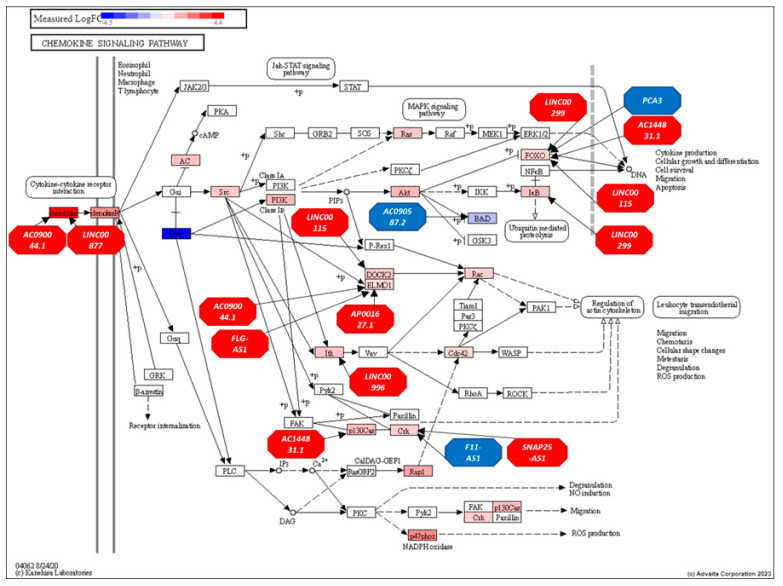
The chemokine signaling pathway (KEGG: hsa04062) illustrates gene regulation differences between AF and EU prostate cancer patients, highlighting the influence of lncRNAs identified through structural equivalence analysis. RED: upregulated and BLUE: downregulated. Genes are presented as rectangles and lncRNAs as octagons.

In the chemokine signaling pathway, 11 out of the 43 structurally equivalent lncRNAs interacted with 10 genes within the pathway, these lncRNAs included *AC090044.1*, *AC090587.2*, *AC144831.1*, *LINC00299*, *LINC00996*, *LINC00115*, *F11-AS1*, *FLG-AS1*, *PCA3*, *AP001627.1,* and *LINC00877*. The mRNAs with which these lncRNAs interacted were *CCL8*, *BAD*, *BCAR1*, *NFKBIB*, *ITK*, *DOCK2*, *CRK*, *FOXO3*, *ELMO1,* and *CXCL12*. Figure 10 displays the chemokine signaling pathway with these structurally equivalent lncRNAs and their interaction with pathway genes.

**Figure 11:**
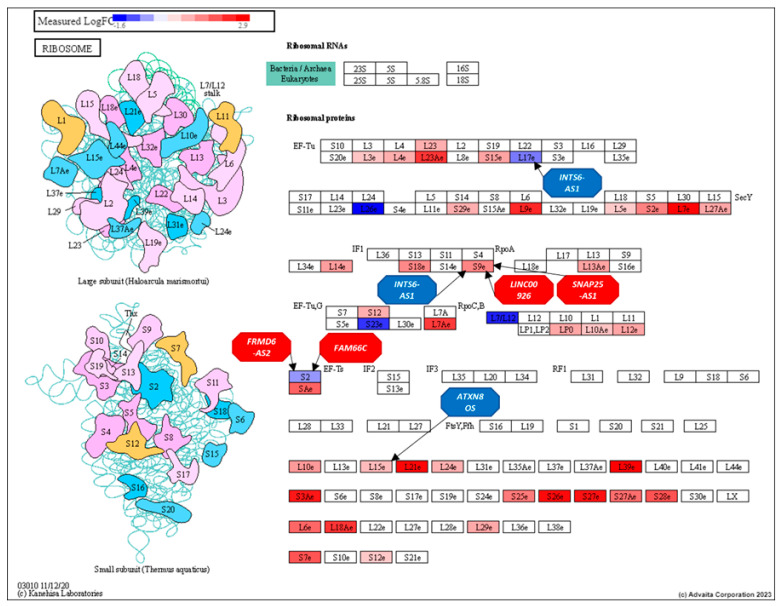
Ribosome pathway (KEGG: 03010) displaying DE gene expression between AF and EU prostate cancer patients, highlighting the influence of specific long non-coding RNAs identified using structural equivalence analyses. Red: upregulated and Blue: downregulated. Genes are presented as rectangles and lncRNAs as octagons.

In the ribosome pathway (Figure 11), six of the 43 lncRNAs identified through centrality analysis interact with four specific genes: *RPS9*, *RPL17*, *RPL15*, and *MRPS2*. These lncRNAs are *LINC00926*, *INTS6-AS1*, *ATXN8OS*, *FRMD6-AS2*, *SNAP25-AS1*, and *FAM66C*. Figure 11 illustrates the ribosome pathway, highlighting these lncRNAs and their interactions with the aforementioned genes.

**Figure 12:**
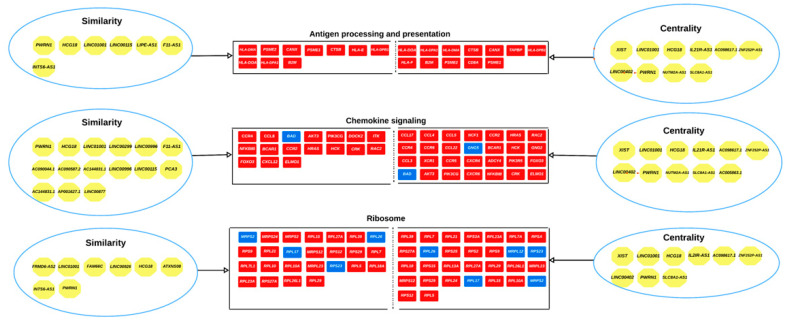
In AF men, several biological pathways were enriched with lncRNAs identified through centrality and structural equivalence metrics. These lncRNAs (organized in circles) influence multiple genes within each pathway (indicated by an arrow), suggesting their significant role in the regulation of these biological processes. These lncRNAs are involved in pathways related to immune responses, inflammation, and cancer progression, suggesting distinct transcriptional programs in AF and indicating their potential impact on disease development and progression. Genes are presented as rectangles and lncRNAs as octagons. Red: upregulated and Blue: downregulated.

Based on centrality and structural equivalence analyses, several long non-coding RNAs (lncRNAs) were identified as influential regulators in three immune-related pathways. LncRNAs with high centrality scores significantly impact these pathways due to their extensive interactions with multiple mRNAs. Conversely, lncRNAs identified through structural equivalence act as micro-influencers within these immune pathways, each contributing uniquely to prostate cancer biology. Figure 12 provides an overview of these lncRNAs and their roles in modulating gene expression within the associated pathways.

### 3.5. lncRNA Differential Expression (Vitamin D Supplementation)

To uncover molecular changes associated with vitamin D supplementation in AF and EU men, two differential expression (DE) analyses were conducted. The first compared AF men receiving vitamin D supplementation to those given a placebo, while the second focused on EU men under the same conditions. To identify significantly DE genes and long non-coding RNAs (lncRNAs) in both racial groups, thresholds of q ≤ 0.4 and with a linear fold change ≥1.5 were applied. This less stringent significance threshold was adopted due to the small sample sizes: four AF men received vitamin D supplementation versus six who were on a placebo, and eight EU men received supplementation versus nine who were on a placebo. This approach aligns with the earlier study on the same patient dataset. In the AF cohort, 711 transcripts were identified as significantly DE (Supplemental Table S11).

**Figure 13:**
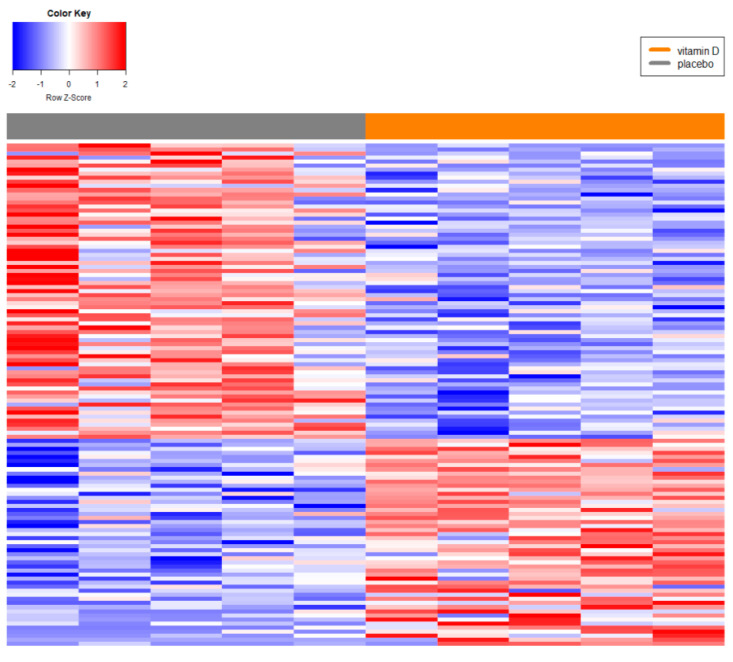
Differential expression of lncRNAs between AF patients who received vitamin D supplementation or a placebo. A total of 124 lncRNAs DE between AF men who received vitamin D supplementation or a placebo are presented. Red and blue boxes indicate relative over- and under expression with respect to a reference calculated as the midpoint between the vitamin D and placebo groups. Only lncRNAs found significant at the level q ≤ 0.4 and with a linear fold change of ≥ 1.5 in the comparison are shown.

No DE transcripts were found in the EU cohort, excluding this group from further downstream analysis. Pathway enrichment analysis of upregulated transcripts (293 in total) revealed significant involvement in immune-related pathways, including CXCR chemokine receptor binding (GO:0045236), chemokine activity (GO:0008009), interleukin-8 receptor binding (GO:0005153), chemokine receptor binding (GO:0042379), and cytokine receptor binding (GO:0005126). Downregulated transcripts (418 in total) were significantly associated with pathways related to cytoskeletal protein binding (GO:0008092), transmembrane transporter binding (GO:0044325), calcium ion binding (GO:0005509), and creatine kinase activity (GO:0004111). These findings suggest that vitamin D supplementation may modulate immune response pathways in AF men with prostate cancer, potentially contributing to observed racial disparities in disease progression. Further research is needed to elucidate the underlying mechanisms and to assess the clinical implications of these molecular changes. Of the 711 DE transcripts at q ≤ 0.4, 124 were categorized as lncRNAs (Figure 13, Supplemental Table S12). I examined the list of transcripts from the AF vs. EU analysis results and AF vitamin D vs. placebo for lncRNA overlap between the two analyses. This identified 34 lncRNAs as overlapping (Figure S18 and Supplemental Table S13).

#### Vitamin D Network Analyses

In analysing DE lncRNAs between AF men receiving vitamin D supplementation or a placebo, interactions with mRNAs were identified. Thirteen lncRNAs—*AC007743.1*, *AC093390.1*, *ADAMTS9-AS2*, *FENDRR*, *LINC00470*, *LINC00607*, *LINC00886*, *LINC01001*, *LINC01082*, *MID1IP1-AS1*, *PCA3*, *PHPN1-AS1*, and *SLC26A4-AS1*—were found to interact with DE mRNA targets (Supplemental Table S14).

Notably, four lncRNAs—*AC007743.1*, *ADAMTS9-AS2*, *LINC01001*, and *PCA3*—were differentially expressed both between AF and EU patients and between AF men receiving vitamin D supplementation versus a placebo. These four lncRNAs share ten mRNA targets: *FLRT2*, *GRIA3*, *PLCL1*, *GSTM5*, *ANK2*, *KCNJ3*, *COL4A4*, *CCDC85A*, *EBF1*, and *CHKB*.

These findings suggest that vitamin D supplementation may modulate specific lncRNA–mRNA interactions in AF men, potentially influencing gene expression patterns associated with prostate cancer progression. Further research is needed to elucidate the functional implications of these interactions and their role in the racial disparities observed in prostate cancer outcomes.

## 4. Discussion

Within the last decade, evidence has been accumulating steadily to suggest that lncRNAs can regulate target mRNAs through a series of specific mechanisms (i.e., scaffolds, decoys, signals etc.) and can play a fundamental role in cancer progression [64,65,66]. While lncRNAs are a relatively new area in cancer research, they have already shown excellent potential for use as biomarkers. The lncRNA *PCA3* was first discovered in 1999 [67]. In 2012 it received FDA approval as a lncRNA based PC biomarker. *PCA3* is detectable in urine, making it non-invasive, an attractive attribute for cancer biomarkers [68]. However previous research has suggested the clinical utility of *PCA3* may be restricted to EU men. Considering this information, the first objective was to identify lncRNAs specific to AF men diagnosed with localized PC and investigate the role these lncRNAs are potentially playing in PC racial differences.

Vitamin D deficiency is considered a risk-factor for the racial differences observed in PC [28]. Individuals of African descent are significantly more prone to severe vitamin D deficiency than individuals of European descent. Studies have shown that AF individuals can have as much as a 15 to 20 times higher chance of having severe vitamin D deficiency in comparison to EU [28]. Based on this information, the second objective of this study was to explore whether vitamin D3 supplementation provided any potentially beneficial effects for AF men diagnosed with localized PC and to observe its effect on lncRNAs and their target mRNAs.

Since the advent of next generation sequencing in the early 2000s, a vast quantity of biological data has been generated, revolutionizing the fields of genomics and transcriptomics. Network biology has emerged as a popular computational approach for the analysis of complex biological systems [14,69]. Almost a decade ago we investigated racial differences in localized prostate cancer using systems biology approaches. This preliminary research found that African men displayed increased gene expression linked to immune response and inflammatory processes, suggesting that these mechanisms may play a role in the more aggressive progression of prostate cancer observed in this group [10]. This study expands on that study with a renewed focus on lncRNAs achieved by incorporating a network biology approach.

We first examined lncRNA expression differences between the AF and EU PC patients. This identified that the lncRNA *AD000090.1* was the most significant downregulated gene (q = 2.27^21^ and linear fold change = −105.6) and *BX255923.1* was the most significant upregulated gene (q = 3.26^−12^ and linear fold change = +26.3) across AF and EU patients. *AD000090.1* has no defined function, however, it has been proposed to regulate hypoxic responses [70]. *BX255923.1* also has no described function. Both lncRNAs are currently uncharacterized in the context of PC.

A key focus of this study was to determine whether lncRNAs influenced mRNA expression and to identify the biological processes impacted by these lncRNAs in AF. The analysis revealed that 142 of the 1283 differentially expressed (DE) lncRNAs in AF interacted with 4158 mRNAs. Notably, 90% of the lncRNAs were excluded as they lacked significant DE mRNA targets, as predicted by the PRAD LongHorn algorithm. The biological interpretation of these 4158 mRNA targets highlighted a recurring theme, with immune response and inflammatory processes emerging as the most significantly associated pathways (e.g., regulation of the immune system and immune response) [10]. Overall, these findings indicate that immune response and inflammatory processes play a role in the prostate cancer disparities observed between AF and EU men, with lncRNAs likely contributing to these differences by regulating genes associated with these pathways.

The next task was to identify which of the DE lncRNAs shared between AF and EU males were the most important in terms of degrees of centrality. Among the 142 differentially expressed (DE) lncRNAs with predicted mRNA targets, 11 stood out for having the highest number of mRNA interactions. Interestingly, the DE lncRNAs with the highest and lowest linear fold changes (*AD000090.1* and *BX255923.1*) were not included in this top group.

*XIST* (X Inactive Specific Transcript) emerged as the lncRNA with the most mRNA interactions, accounting for 53% of the total connections. *XIST* was downregulated in AF men relative to EU men (−1.9 linear fold change). *XIST* is involved in immune response and cancer growth. Recently *XIST* has been described as a tumor suppressor transcript in multiple cancer types and a potential biomarker in PC [71]. Lower levels of *XIST* are likely linked to more aggressive tumor behavior and reduced survival rates, suggesting a pivotal role in mediating racial differences in gene expression between AF and EU prostate cancer patients. Detailed evaluation of the top 11 lncRNAs, each with the highest number of mRNA targets, highlights the intricate nature of lncRNA–mRNA interactions. Notably, all 11 top-ranked lncRNAs exhibited the capacity to modulate the expression of their associated mRNA targets, driving either upregulation or downregulation of gene expression.

The second lncRNA with the greatest number of mRNA interactions was *LINC01001* [long intergenic non-protein-coding RNA 1001], a transcript currently not associated with PC. This transcript was upregulated and functions primarily as a transcriptional factor decoy to help regulate transcription. Decoy lncRNAs typically function to suppress transcription by hindering the activity and usage of distinct molecules such as miRNAs, transcriptional binding proteins, and transcriptional factors, however, they have been found to positively regulate gene expression [72]. Prior research identified *LINC01001* as differentially upregulated and a prognostic biomarker in lung adenocarcinoma [73]. In 2021, *LINC01001* was found to promote the progression of Crizotinib resistant non-small-cell lung cancer [74]. *LINC01001* was found to be DE in AF compared to EU men [62]. Further research is necessary to fully comprehend the mechanism by which *LINC01001* affects AF PC patients.

The third ranked lncRNA in terms of network centrality was *HCG18* [HLA Complex Group 18], an lncRNA related to the histocompatibility complex vital for the normal functioning of the immune system [75]. The histocompatibility complex plays a crucial role in enabling the immune system to differentiate between the body’s own proteins and those produced by foreign entities [76]. We noted that *HCG18* was positively regulated in AF men and regulated greater than 200 gene targets with multi-functional processes. Recently, *HCG18* was classified as a cancer-related lncRNA and identified as significantly upregulated in colorectal cancer tissues and cell lines [77]. Further studies have identified *HCG18* as downregulated in bladder cancer tissues and cell lines, suggesting its potential as a prognostic lncRNA. Its mechanism of action involves cooperation with *NOTCH1*, which can act both as an oncogene and tumor suppressor [78]. Interestingly, *NOTCH1* was not significantly differentially expressed in AF, nor were any members of the NOTCH family found to be regulated by *HCG18* in this study. In 2021, Chen et al. investigated lncRNAs in the context of PC bone metastasis and noted that *HCG18* and its target mRNAs were associated with tumor-related immune cells, particularly M2 macrophages [79]. M2 macrophages are a subset of macrophages involved in tissue repair, immune regulation, and anti-inflammatory responses. They are part of the macrophage polarization spectrum, where macrophages adopt different functional states (M1 or M2) in response to environmental cues [80]. They play a role in promoting immune regulation while maintaining anti-inflammatory activity [81]. M2 macrophages influence prostate cancer progression through the activation of both the NF-κB and JAK-STAT signaling pathways [82]. The NF-κB pathway is critical in cancer development and progression, facilitating tumor cell proliferation and angiogenesis [83]. Similarly, the JAK-STAT pathway is a key driver of cancer progression and has been implicated in various cancer types and autoimmune diseases [84].

*IL21R-AS1* is an antisense RNA to the protein-coding gene the Interleukin 21 Receptor (IL21R), yet with no documented biological function. Nevertheless, immunodeficiency is one condition caused by loss of function mutations in the *IL21* and *IL21R* genes [85]. We observed decreased expression of *IL21R-AS1* in AF patients with no statistically significant alteration in the expression levels of *IL21R* itself, indicating that the effect is specific for expression of the lncRNA. This suggests that the immune effects associated with *IL21R-AS1* expression are primarily driven by its own activity and interactions, rather than being a secondary consequence of *IL21R* expression and its effects.

*AC098617.1* encodes a novel transcript and an antisense RNA to *TMEFF2* [Transmembrane Protein with EGF-like and two Follistatin-like domains]. The biological function of *AC098617.1* is currently unknown; however, its associated gene, *TMEFF2*, is recognized as an androgen-regulated tumor suppressor in prostate cancer [86]. *TMEFF2* is over-expressed in primary forms and castration-resistant forms of PC [87]. *AC098617.1* was significantly upregulated in this study in AF men and functions as a co-factor lncRNA in its interactions with its gene targets. Co-factor lncRNAs generally function by altering transcriptional factor promotor interactions. *TMEFF2* was not significantly DE, suggesting that the effect is specific for the expression of *AC098617.1* and not *TMEFF2.* This indicated that *AC098617.1* acts alone in regulating its target mRNAs without the need for significant *TMEFF2* expression. *AC098617.1* was identified as significantly DE in African men in both the Yuan et al. (2020) and Rayford et al. (2021) studies [61,62]. Further research is needed to elucidate the molecular function of *AC098617.1* in the context of prostate cancer in AF patients.

*ZNF252P-AS1* was the sixth highest-ranked lncRNA in terms of the number of genes it regulates [ZNF252P Antisense RNA 1]. Currently, *ZNF252P-AS1* has no defined biological role. Interestingly, the *ZNF252P* transcript is classified as a transcribed unprocessed pseudogene [88,89]. A recent study identified *ZNF252P* as downregulated in multiple cancer types including PC [90]. Another important aspect related to this lncRNA is its chromosomal location, 8q24. *ZNF252P-AS1* is directly located inside this locus, which is recognized as a cancer susceptibility locus and particularly important for cancer in patients of African ancestry [91]. The protein encoded by *ZNF252P* is a putative uncharacterized protein. *ZNF252P-AS1* is significantly downregulated in AF men and principally acts as a co-factor. Interestingly, *ZNF252P* is not significantly DE, suggesting that the effect of *ZNF252P-AS1* is unique to AF men in PC.

*LINC00402* [long intergenic non-protein-coding RNA 402] was identified as significantly upregulated in AF men and acts as a multi-functional lncRNA, regulating over 90 genes. *LINC00402* has no described biological function and remains uncharacterized in PC. Further research is required to explore the mechanism of action of *LINC00402* and its role in racial differences in PC.

*PWRN1* [Prader–Willi region non-protein-coding RNA 1] has been previously correlated with regulating gastric cancer, however, it is not associated with PC. *PWRN1* is located within the Prader–Willi syndrome region of chromosome 15. Prader–Willi syndrome is a neurogenetic disease which is caused by loss of expression of paternal genes in the 15q11.2-q13 region [92]. We noted that *PWRN1* was acting as a multi-functional lncRNA to support up- and down-regulation of target genes.

*NUTM2A-AS1* is an antisense RNA to the protein-coding gene *NUTM2A* [Nut [Nuclear Testis Protein) Family Member 2A]. Previously, research has shown that *NUTM2A-AS1* is involved in the progression of gastric cancer tumorigenesis [93]. *NUTMA-AS1* was identified as ubiquitously expressed in 14 human tissues, including PC tissue [94]. *NUTM2A* was not identified as significantly DE in AF men, suggesting the effect is specific to *NUTM2A-AS1*.

The remaining lncRNAs, ranked based on centrality, were *SLC8A1-AS1* and *AC005863.1*. *SLC8A1-AS1* is an antisense RNA to the gene *SLC8A1* (Solute Carrier Family 8 Member A1). *SLC8A1-AS1* was upregulated in AF men and operating as a multi-factorial lncRNA to regulate target mRNAs. To date, no research has linked *SLC8A1-AS1* to PC. While its associated protein-coding gene, *SLC8A1*, was reported as significantly downregulated in a previous bioinformatics study on prostate cancer, it was not significantly differentially expressed in this study [95]. Similarly, *AC005863.1* is a novel transcript with no known connection to cancer. It was upregulated in AF and, like the other top-ranked 10 lncRNAs, functions as a multi-functional lncRNA involved in regulating target mRNAs.

A total of 43 lncRNAs in AF men were identified as structurally equivalent (11 of which were the top-ranking lncRNA identified as central and described above). Using all the mRNA targets of these 32 additional lncRNAs identified immune and inflammatory related processes including “leukocyte activation” and “T-cell activation”, which were both over-represented processes identified in AF men in our earlier study [24]. Of particular interest in this group of lncRNAs was *AC084125.4,* which is located on 8q24, a susceptibility locus for multiple cancer types including PC [58]. Currently, *AC084125.4* is uncharacterized and is not associated with any type of cancer.

Pathways found to be over-represented in AF men included antigen processing and presentation (APP) (KEGG: 04612), chemokine signaling (KEGG: 04062), and ribosome (KEGG: 03010). By combining the target genes of the top 11 ranked lncRNAs (centrality) and the synergistically acting lncRNAs (structurally equivalence) in AF men, we demonstrated that many of these lncRNAs interacted with genes belonging to these pathways. The APP biological pathway is responsible for facilitating the direct interaction between cancer and the adaptive immune system [96]. In our original study, “lymphocyte activation and T-cell activation” were identified as an inflammatory related impacted pathways in AF men with significant elevated gene expression compared to EU men. A group of 15 lncRNAs were found to cooperatively influence gene expression of genes belonging to the APP biological pathway. These lncRNAs included *XIST*, *LINC01001*, *HCG18*, *IL2IR-AS1*, *AC098617.1*, *ZNF252P-AS1*, *LINC00402*, *PWRN1*, *NUTM2A-AS1*, *SLC8A1-AS1*, *LINC00115*, *F11-AS1*, *LIPE-AS1*, *SNAP25-AS1,* and *INTS6-AS1*. These results suggest that lncRNAs are contributing to the regulation of this pro-inflammatory response.

The second biological pathway found to be over-represented in AF men was chemokine signaling [97]. A group of 22 lncRNAs cooperatively influenced genes in the chemokine signaling biological pathway. These lncRNAs included *XIST*, *LINC01001*, *HCG18*, *IL2IR-AS1*, *AC098617.1*, *ZNF252P-AS1*, *LINC00402*, *PWRN1*, *NUTM2A-AS1*, *SLC8A1-AS1*, *AC005863.1*, *AC090044.1*, *AC090587.2*, *AC144831.1*, *LINC00299*, *LINC00996*, *LINC00115*, *F11-AS1*, *FLG-AS1*, *PCA3*, *AP001627.1*, and *LINC00877*.

A second objective of this study was to examine whether vitamin D3 supplementation could alter mRNA and lncRNA expression in PC patients of AF and EU descent and determine if it was beneficial to AF men. Vitamin D deficiency may be a key contributor to racial differences in PC due to many AF men being vitamin D deficient and having increased incidence and mortality rates for PC [98].

We noted that vitamin D altered lncRNA and mRNA expression only in AF patients. This result suggests that vitamin D was only influential for AF men and had little or no effect on mRNA and lncRNA expression in EU patients. This is in line with the findings previously reported by us where we observed enriched anti-inflammatory-related GO terms. These included inflammatory processes related to chemokine activity, chemokine receptor binding, inflammatory response, and cell–cell signaling [24,97,99].Inflammation is a crucial factor in increasing the risk of PC, metastasis, and therapeutic resistance [100,101]. This result suggests that vitamin D is beneficial for patients of AF descent [98,102,103,104,105]. At the lncRNA level, we noted 124 DE lncRNAs in AF patients who received vitamin D supplements, suggesting that vitamin D influences lncRNA expression in AF men. The following lncRNAs (*AC007743.1*, *ADAMTS9-AS2*, *LINC01001,* and *PCA3)* were DE in common between AF and EU men and AF men who received vitamin D supplements relative to a placebo, suggesting that they are the most important lncRNAs regulated by vitamin D supplementation and are beneficial to AF men in regulating anti-inflammatory processes. Zhao et al., 2022, identified *AC007743.1* as one of six lncRNAs that are associated with necroptosis, and which are independent prognostic predictors of clear cell renal cell carcinoma [106]. In 2023, Cao et al. highlighted *AC007743.1* as a prognostic lncRNA for colon cancer [107]. We observed that AC007743.1 was upregulated in AF men (q ≤ 0.1 and fold change ≥ 1.5), however, in AF men who received vitamin D supplements, *AC007743.1* was downregulated (q ≤ 0.4 and fold change of ≥ 1.5), suggesting the potential anti-inflammatory effects of vitamin D supplementation in AF PC patients.

*ADAMTS9-AS2* (ADAM Metallopeptidase with Thrombospondin Type 1 Motif 9 Antisense RNA 2) is categorized as a tumor suppressor lncRNA for a variety of cancer types [108]. Abnormal expression of *ADAMTS9-AS2* has been linked to cancer cell proliferation, invasion, migration, and the inhibition of cell death. Previous research has linked *ADAMTS9-AS2* with the PI3K/Akt signaling pathway. The Phosphatidylinositol-3-kinase (PI3K)/AKT/mammalian target of the rapamycin (mTOR) signaling (PI3K-Akt) pathway is an important biological pathway that plays a role in the progression of cancer. Vitamin D is a potential therapeutic option in the treatment of non-small-cell lung cancer through its ability to inactivate the PI3K-Akt signaling pathway [109]. Further research is required to determine the link between lncRNA *ADAMTS9-AS2*, vitamin D supplementation, and the PI3K-Akt signaling pathway.

*LINC01001* was identified earlier in our study as the second-ranked lncRNA to be interacting with the greatest number of mRNA targets. *PCA3* was first observed as being highly expressed in PC tumors by Bussemakers et al. in 1999 [110]. Since then, *PCA3* has been developed as a urine-based diagnostic biomarker for PC. *PCA3* was downregulated in AF patients but, with a short course of vitamin D supplementation, its expression was upregulated. The observation that *PCA3* was downregulated in AF men was an expected result. *PCA3* is categorized as a PC biomarker, however, its utility as a reliable biomarker in AF men has been questioned. O’Malley et al., 2017, investigated whether the measurement of *PCA3* held utility for the detection of PC in both AF and EU men [111]. This study noted that *PCA3* did improve clinical utility but only for EU men, suggesting that it is not an ideal biomarker for AF men [111]. Research is limited at present as to whether *PCA3* does in fact promote progression of PC. If *PCA3* does play a role in the progression of PC, this finding can be taken as a controversial result, highlighting the need for AF-specific lncRNA biomarkers. The finding that *PCA3* expression was upregulated in response to vitamin D supplementation suggests that *PCA3* is a suitable biomarker in EU men (who are typically vitamin D sufficient) and not in AF men who are vitamin D sufficient.

In total, 63 mRNA targets were observed for these lncRNAs that were uniquely DE in AF men who received vitamin D supplementation. A systems-level analysis revealed enriched GO terms related to cell junction and calcium related functions [112]. Vitamin D and calcium are interconnected, and both are vital for normal bone health. Vitamin D sufficiency is necessary for promoting calcium absorption [105]. These results suggest that the lncRNAs *AC007743.1*, *ADAMTS9-AS2*, *LINC01001*, and *PCA3* are regulated by vitamin D and subsequently regulate mRNAs associated with calcium absorption.

We observed that calcium and cell-junction-related GO terms were shared in the comparison between AF and EU men and AF men who received vitamin D supplementation or a placebo. Further analysis revealed that the lncRNA *ADAMTS9-AS2* interacted with mRNAs related to both calcium function and cell junction function, highlighting the importance of both in AF men, and particularly those deficient in vitamin D. Taken together, these results suggest the lncRNAs *AC007743.1*, *ADAMTS9-AS2*, *LINC01001,* and *PCA3* are regulated by supplementation and are important in AF men deficient in vitamin D. *LINC01001* was the second-ranked central lncRNA in AF men. Collectively, these results suggest that vitamin D supplementation may represent a key mechanism to address racial disparities in PC via its role in modulating lncRNA regulation of mRNA targets in PC.

We recognize that the sample size is a limitation of this study. To address this, future studies will aim to increase the number of enrolled participants by extending RNA-seq analyses to include single-core prostate biopsy samples obtained prospectively. The RNA-seq results reported here were generated from tissue samples weighing less than 50 mg, comparable to a single-core biopsy. Tumor stage and grade are critical factors that can influence gene expression profiles and lncRNA regulation, potentially contributing to differences in molecular pathways across various pathological stages. The predominance of T2/T3 staging of the tumors examined in this study might limit the generalizability of the findings to other staging groups, e.g., T4. In future studies, participants will also be stratified based on race, serum vitamin D levels, PSA levels, Gleason scores, and supplementation status. These expanded clinical studies will help validate the hypothesis that the prostate, at the molecular level, serves as a “sentinel” organ for health disparities.

Another limitation was the reliance on pre-generated lncRNA–mRNA interactions from the LongHorn PRAD algorithm to construct lncRNA-target-gene regulatory networks. Future studies will develop custom interaction databases using experimental datasets or publicly available resources like ENCODE and FANTOM. Experimental validation, such as RNA immunoprecipitation sequencing (RIP-Seq) or CRISPR-based perturbation, will be employed to confirm predicted interactions and uncover novel ones. Integrating multi-omics data, including transcriptomics, proteomics, and epigenomics, will, in the future, provide a broader perspective on regulatory relationships. Refining the LongHorn PRAD algorithm or utilizing alternative prediction tools, like LncBase or RNAInter, could enhance robustness, while network inference approaches, such as weighted gene co-expression network analysis (WGCNA), could construct de novo interaction networks directly from RNA-seq data. These strategies would reduce dependence on pre-existing datasets and improve the accuracy and specificity of lncRNA-target-gene regulatory networks.

## 5. Conclusions

LncRNAs contribute to racial differences between AF and EU men in terms of the effects of PC. LncRNAs regulating immune response and inflammatory processes potentially contribute to increased disease severity in AF men. LncRNAs regulating the greatest number of mRNA targets in AF men were multi-factorial in their regulation of target mRNAs, highlighting the complexity of their role in PC. Most of the top-ranked lncRNAs with the highest number of mRNA targets had not been previously linked to prostate cancer (PC). Moreover, their specific biological functions remain undefined, underscoring the need for further investigation and their potential as biomarkers and therapeutic targets for PC in African men. Vitamin D influenced mRNA and lncRNA expression exclusively in African men in this patient cohort. At the transcriptomic level, it demonstrated anti-inflammatory effects by modulating chemokine activity and the inflammatory response. This finding aligns with the initial mRNA analysis conducted by Hardiman et al., 2016. Additionally, vitamin D was found to regulate lncRNAs associated with the mRNAs involved in calcium signaling and cell junction functions, both critical factors in cancer metastasis.

## Figures and Tables

**Table 1:** Top-ranked GO terms enriched in AF patients by DE lncRNA target mRNAs. Over-representation analysis of the DE lncRNA target mRNAs in AF patients using the Gene Ontology Biological Process database.

Gene Ontology Biological Process	*p*-Value	q-Value FDR B and H
Regulation of immune system process	1.686 × 10^−15^	6.107 × 10^−12^
T-cell migration	3.414 × 10^−3^	3.264 × 10^−2^
Regulation of immune response	2.456 × 10^−12^	1.776 × 10^−9^
Leukocyte activation	1.242 × 10^−12^	1.125 × 10^−9^
Regulation of defense response	4.105 × 10^−5^	8.801 × 10^−4^

**Table 2:** Chromosomal locations of the top-ranking lncRNAs.

Top-Ranking lncRNAs	Number of mRNA Interactions	Chromosome Location
*XIST*	574	Xq13.2
*LINC01001*	317	11p15.5
*HCG18*	243	6p22.1
*IL21R-AS1*	233	16p12.1
*AC098617.1*	181	2q32.3
*ZNF252P-AS1*	114	8q24.3
*LINC00402*	93	13q22.1
*PWRN1*	92	15q11.2
*NUTM2A-AS1*	90	10q23.2
*SLC8A1-AS1*	87	2p22.1
*AC005863.1*	87	17p12

**Table 3:** lncRNAs commonly identified across these studies and our results. Information on whether the lncRNAs were differentially expressed (DE) in those studies, the associated FDR values, and whether they were upregulated or downregulated is included.

Article	LncRNA	Differentially Expressed	FDR	Up- or Downregulated
Yuan et al., 2020 [61]	*AC098617.1*	Yes	0.01	Not defined
Yuan et al., 2020 [61]	*LINC00402*	Yes	0.01	Not defined
Yuan et al., 2020 [61]	*SLC8A1-AS1*	Yes	0.01	Not defined
Yuan et al., 2020 [61]	*AC005863.1*	Yes	0.01	Not defined
Rayford et al., 2021 [62]	*LINC01001*	Yes	0.02	Not defined
Rayford et al., 2021 [62]	*AC098617.1*	Yes	0.02	Not defined

**Table 4:** LncRNA co-occurrence between lncRNAs designated as A and B. Results were generated from CbioPortal.

lncRNA (A)	lncRNA (B)	Neither	A Not B	B Not A	Both	Log_2_ Odds Ratio	*p*-Value	q-Value	Tendency
*XIST*	*IL21R-AS1*	4000	38	14	4	>3	<0.001	<0.001	Co-occurrence
*HCG18*	*ZNF252P-AS1*	3715	54	272	15	1.924	<0.001	0.001	Co-occurrence
*NUTM2A-AS1*	*SLC8A1-AS1*	3967	72	13	4	>3	<0.001	0.003	Co-occurrence
*IL21R-AS1*	*NUTM2A-AS1*	3965	15	73	3	>3	0.004	0.038	Co-occurrence

**Table 5:** Chromosomal locations of the lncRNAs identified using structural equivalence analysis.

lncRNAs Identified Using Structural Equivalence Analysis	Chromosome Location
*AC104024.1*	17p11.2
*AC084125.4*	8q24.3
*LINC00877*	3p13
*DNM3OS*	1q24.3
*LINC00539*	13q12.11
*ATP1B3-AS1*	3q23
*FGF13-AS1*	Xq26.3
*AC107079.1*	2q37.3
*GK-AS1*	Xp21.2
*COL4A2-AS1*	13q34
*FRMD6-AS2*	14q22.1
*HIF1A-AS2*	14q23.2
*AP001627.1*	3q13.12
*LINC00882*	3q13.12
*LINC00987*	12p13.31
*ATXN8OS*	13q21.33
*AC090587.2*	10q24.2
*PCA3*	9q21.2
*PCCA-AS1*	13q32.3
*RAI1-AS1*	17p11.2
*LINC01068*	13q31.1
*LINC00887*	3q29
*HLCS-IT1*	21q22.13
*DDX11-AS1*	12p11.21
*AC144831.1*	17q25.3
*LINC00299*	2p25.1
*LINC00115*	1p36.33
*AP000439.2*	11q13.3
*FAM66C*	12p13.31
*HPN-AS1*	13q13.11
*LINC00313*	21q22.3
*LUCAT1*	5q14.3
*LINC00926*	15q21.3
*ZBTB20-AS4*	3q13.31
*LINC00494*	20q13.13
*CAMTA1-IT1*	1p36.23
*MIR497HG*	17p13.1
*LIPE-AS1*	19q13.2
*FLG-AS1*	1q21.3
*SLC8A1-AS1*	2p22.1
*SNAP25-AS1*	20p12.2
*F11-AS1*	4q35.2
*INTS6-AS1*	13q13.3

## Data Availability

The data that support the findings of this study are available at the National Center for Biotechnology Information (NCBI) Gene Expression Omnibus (GEO) database; accession number GSE189209, https://www.ncbi.nlm.nih.gov/geo/query/acc.cgi?acc=GSE189209, accessed on 3 December 2024.

## Supplementary Materials

The following supporting information can be downloaded at: https://www.mdpi.com/article/10.3390/genes16020229/s1. References [113,114,115,116,117,118,119,120] are cited in Supplementary Materials.
