# Supplementary material for: Unveiling Racial Disparities in Localized Prostate Cancer: A Systems-Level Exploration of the lncRNA Landscape
Source: Genes (Basel). 2025 Feb 17;16(2):229. doi: 10.3390/genes16020229 (PMC11855151; doi:10.3390/genes16020229)
Supplement: Supplementary file 1 [file genes-16-00229-s001.zip › Morgan et al., 2025_Supplemental Material_submission_revised.pdf]

Supplemental Materials

Unveiling Racial Disparities in Localized Prostate Cancer: A Systems-Level Exploration of the lncRNA Landscape

Rebecca Morgan[1], E Starr Hazard[2], Stephen J Savage[3,4],
Chanita Hughes Halbert[5,6], Sebastiano Gattoni-Celli[4,7], Gary Hardiman[1,8]

Affiliations

- [1]Faculty of Medicine, Health and Life Sciences, School of Biological Sciences, Institute for Global Food Security (IGFS), Queen's University Belfast, (QUB), Belfast BT9 5DL, UK.
- [2]Academic Affairs Faculty, Medical University of South Carolina (MUSC), Charleston, SC 29425, USA.
- [3]Department of Urology, Medical University of South Carolina (MUSC), Charleston, SC 29425, USA.
- [4]Ralph H. Johnson VA Medical Center, Charleston, SC 29425, USA.
- [5]Department of Population and Public Health Sciences, University of Southern California, Los Angeles, CA 90033, USA.
- [6]Norris Comprehensive Cancer Center, University of Southern California, Los Angeles, CA 90033, USA.
- [7]Department of Radiation Oncology, Medical University of South Carolina (MUSC), Charleston, SC 29425, USA.
- [8]Department of Medicine, Medical University of South Carolina (MUSC), Charleston, SC 29425, USA.

Supplementary Materials and Methods

1.0 LncRNA Systems Biology Analyses.

The top ranking lncRNAs (AF vs EU) identified using the centrality metric were analysed using additional tools and methods which are described below as shown in Figure 2.

1.2 Association Analyses.

DE results from other RNA-seq studies investigating the genomic and/or transcriptional differences of AF and EU PC patients were utilized to determine whether any of the top 11 ranking lncRNAs (AF vs EU) were also significantly DE in these of these studies. DE studies which were explored included 1. Yuan *et al.*, 2020 which investigated lncRNA and mRNAs between AF and EU men using RNA-seq data from TCGA PRAD (57 AF, 413 EU) [1]. 2. Rahmatpanah *et al.*, 2021 study of RNA-seq of 45 Atlanta VA Medical Center PC patients between AF and EU descent (15 AF, 30 EU) [2] and lastly, 3. Rayford, et al., 2021 which explored DE between AF and EU PC patients using both TCGA and another PC cohort (596 AF, 556 EU)[3].

1.3 CATrapid Omics. CatRapid omics was used to predict potential protein interactions of the top ranked lncRNAs (AF vs EU)[4]. Exon transcript fragments of lncRNAs were obtained from ENSEMBL, prioritizing those with GENCODE basic membership, which ensures at least one transcript per gene regardless of biotype. Flanking sequences at either end of the transcript were set to value 0, as current evidence indicates that many lncRNAs lack conserved flanking regions [5].

1.4 cBioPortal. Each top ranking lncRNA (AF vs EU) was queried within cBioPortal to determine whether they appeared in previous PC studies present in the TCGA [6,7]. Particular attention was given to lncRNAs found to be co-expressed (i.e., co-occurrence) between PC studies. PC studies queried are presented in Table 1.

1.5 Structural equivalence of top 11 ranking central lncRNAs.

The structural equivalence metric was applied to the top 11 ranking lncRNAs and their target mRNAs [8]. This enabled an assessment of the structural similarity among the top ranking lncRNAs by examination of their interactions with shared target mRNAs.

1.6 Structural equivalence analysis of all lncRNAs. The structural equivalence metric was applied to the differentially expressed (DE) transcripts between African (AF) and European (EU) populations, as identified by DESeq2. Intersections were created using significantly DE lncRNAs, significantly DE mRNAs, and the LongHorn PRAD lncRNA-mRNA algorithm results, illustrated in Figure 1. Applying the structural equivalence metric to these interactions enabled the identification of functional similarities among lncRNAs based on shared mRNA targets.

Supplemental Figures

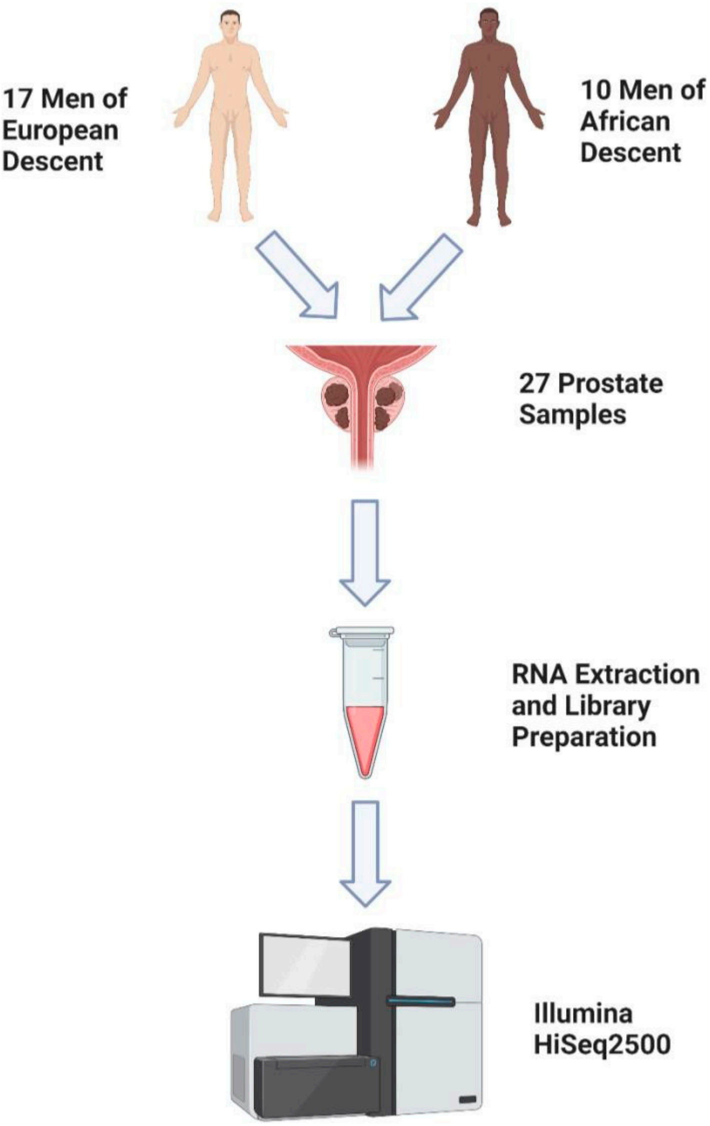

Figure S1. **Collection of RNA-seq data. 27 patients (17 EU, 10 AF) diagnosed with localized PC were recruited for the study.** Patients underwent a medically indicated prostatectomy for treatment of their cancer. Prostate tissue samples were collected from each patient and total RNA collected. Total RNA was then used to prepare RNA-seq libraries and high-throughput sequencing was performed using an Illumina HiSeq2500 sequencing machine as described [10].

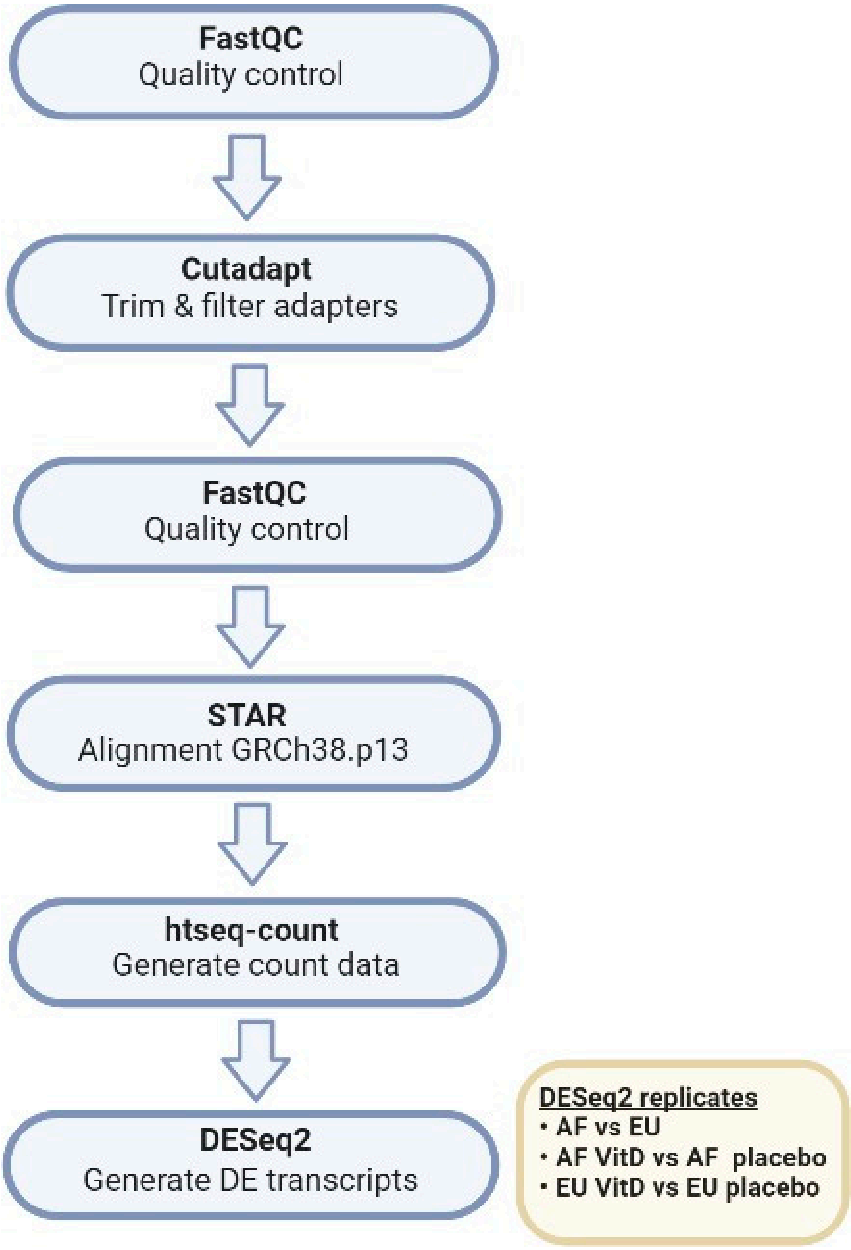

Figure S2A. Schematic outlining RNA-seq pre-processing and DESeq2 analyses. FastQC was used to generate quality reports on individual fastq files. Cutadapt trimmed Illumina library adapter sequences identified by FastQC. FastQC was re-run to determine whether adapter sequences had been successfully removed. The STAR aligner was subsequently used to align the fastq files to the ENSEMBL GRCh38.p13 human genome build. Htseq-count generated count data for all transcripts per patient and DESeq2 was utilized to measure differential expression in specific comparisons.

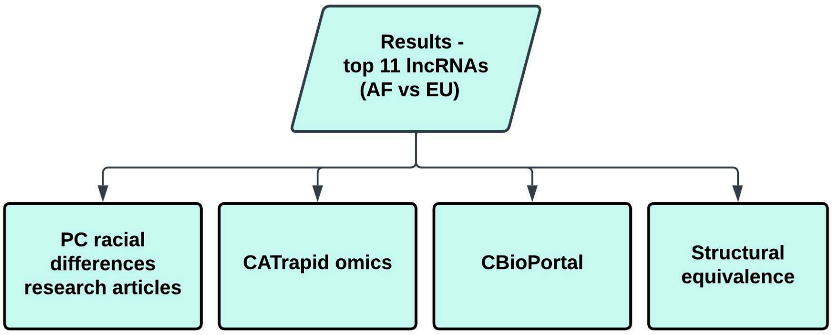

Figure S2B. Workflow outlining downstream analyses of the top 11 ranking lncRNAs.

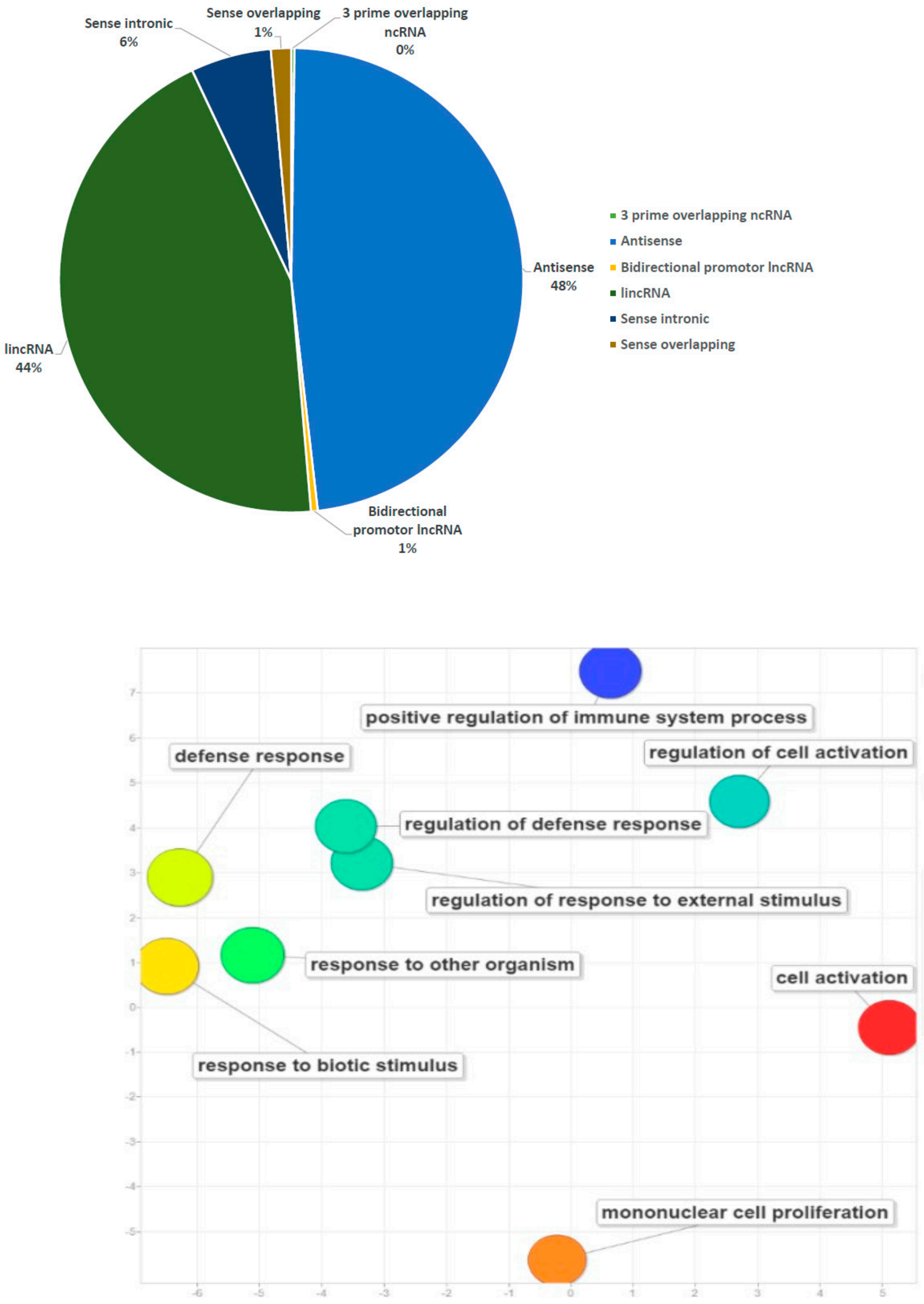

Figure S3. Upper Panel: Classification of the lncRNAS. Bottom Panel: REViGO plot of semantically similar GO terms. Scatterplots displaying clusters of semantically similar GO biological processes in AF patients using the DE lncRNA target mRNAs as input.

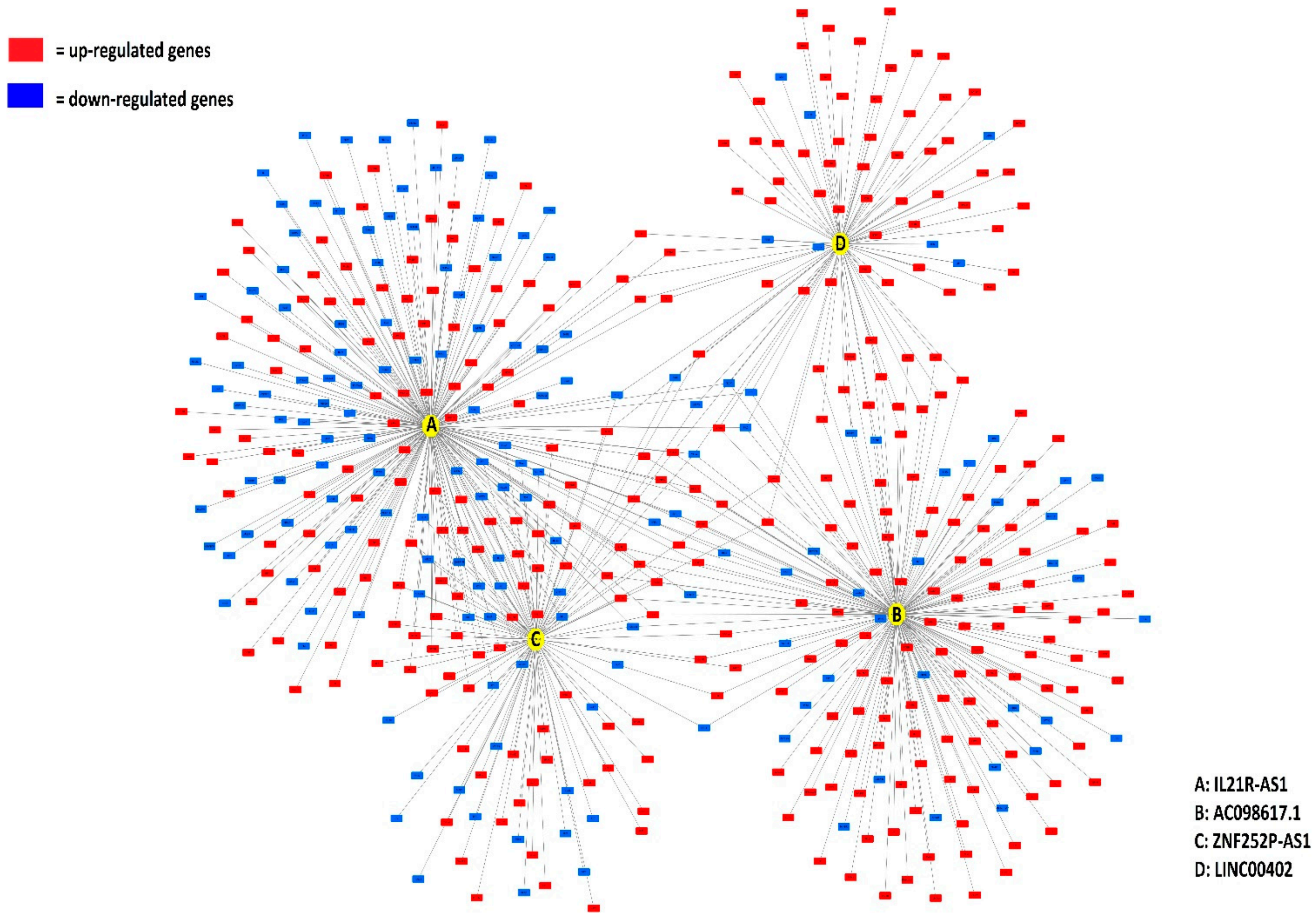

Figure S4. Interactions between *IL21R- AS1*, *AC098617.1*, *ZNF252P-AS1* and *LINC00402* with their mRNA targets. Nodes represent the mRNA targets, with RED indicating upregulated expression and BLUE indicating downregulated expression. Edges depict the interactions between the specified non-coding RNAs and their corresponding mRNA targets, highlighting potential regulatory pathways.

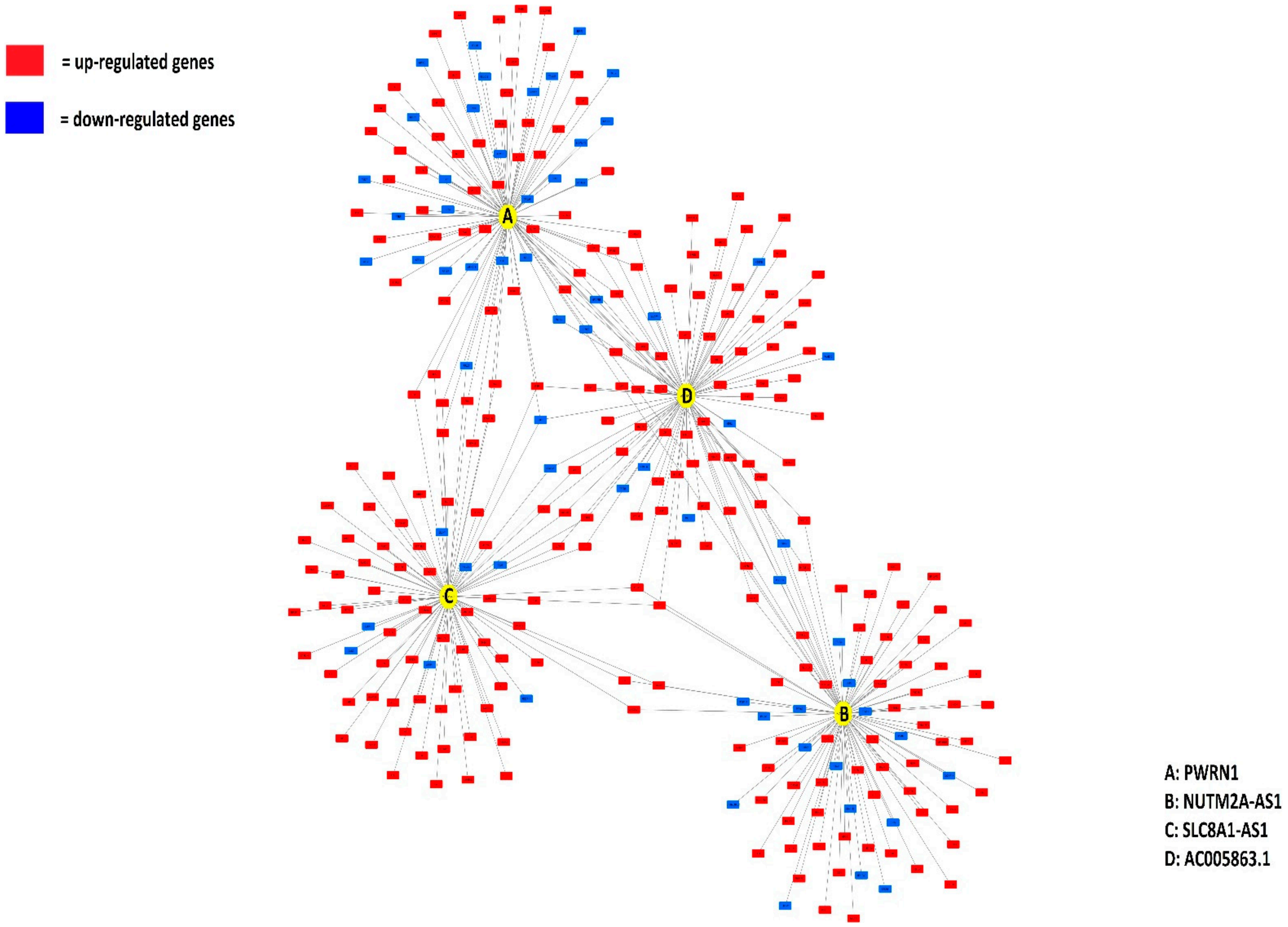

Figure S5. Interactions between *PWRN1, NUTM2A-AS1, SLC8A1-AS1*, and *AC005863.1* with their mRNA targets. Nodes represent the mRNA targets, with RED indicating upregulated expression and BLUE indicating downregulated expression. Edges depict the interactions between the specified non-coding RNAs and their corresponding mRNA targets, highlighting potential regulatory pathways.

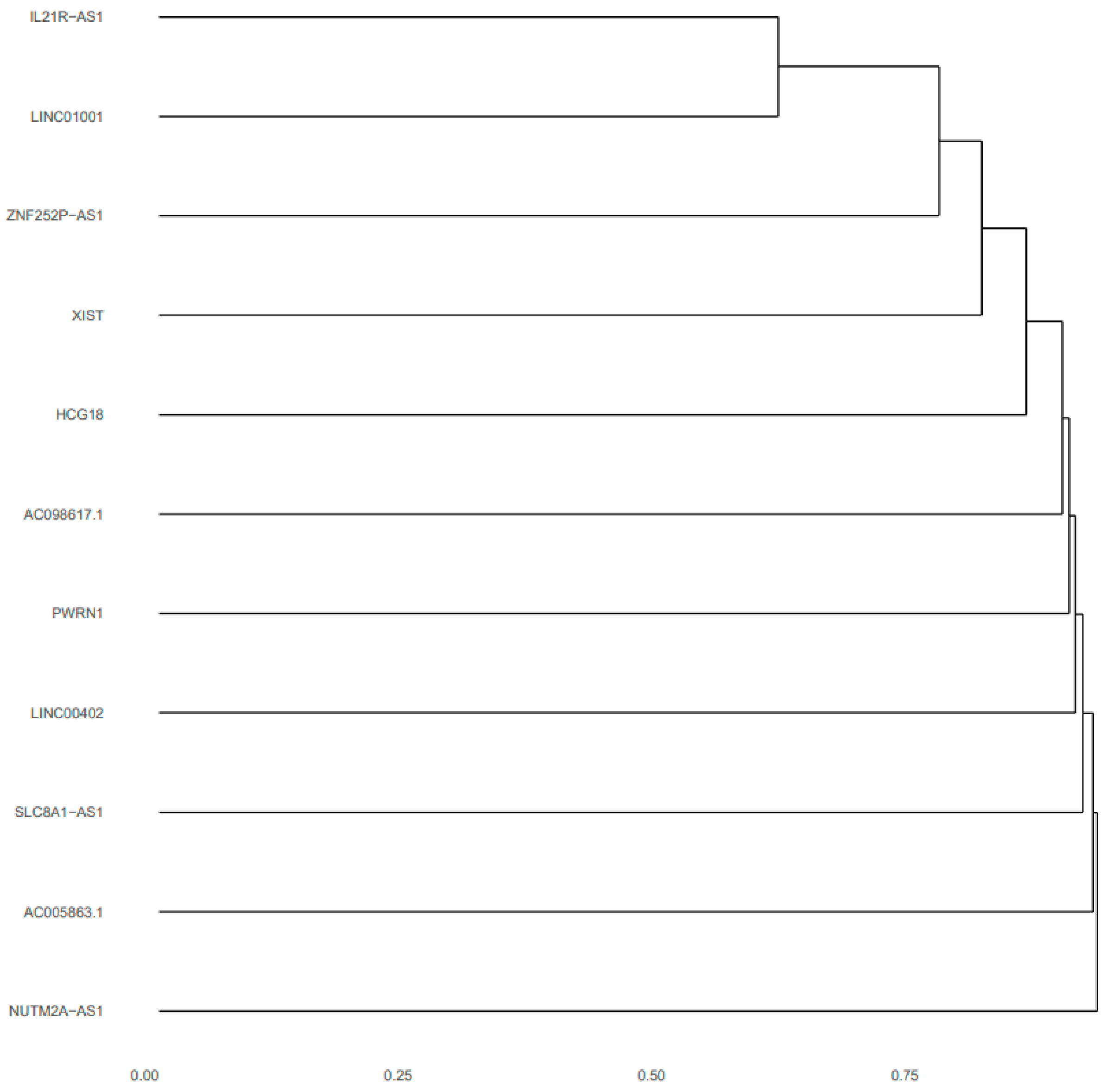

Figure S6. Structural equivalence of the top ranking lncRNAs. Dendrogram plot of the top ranking lncRNAs based on the number of shared mRNA targets. The x-axis represents the Jaccard index scores.

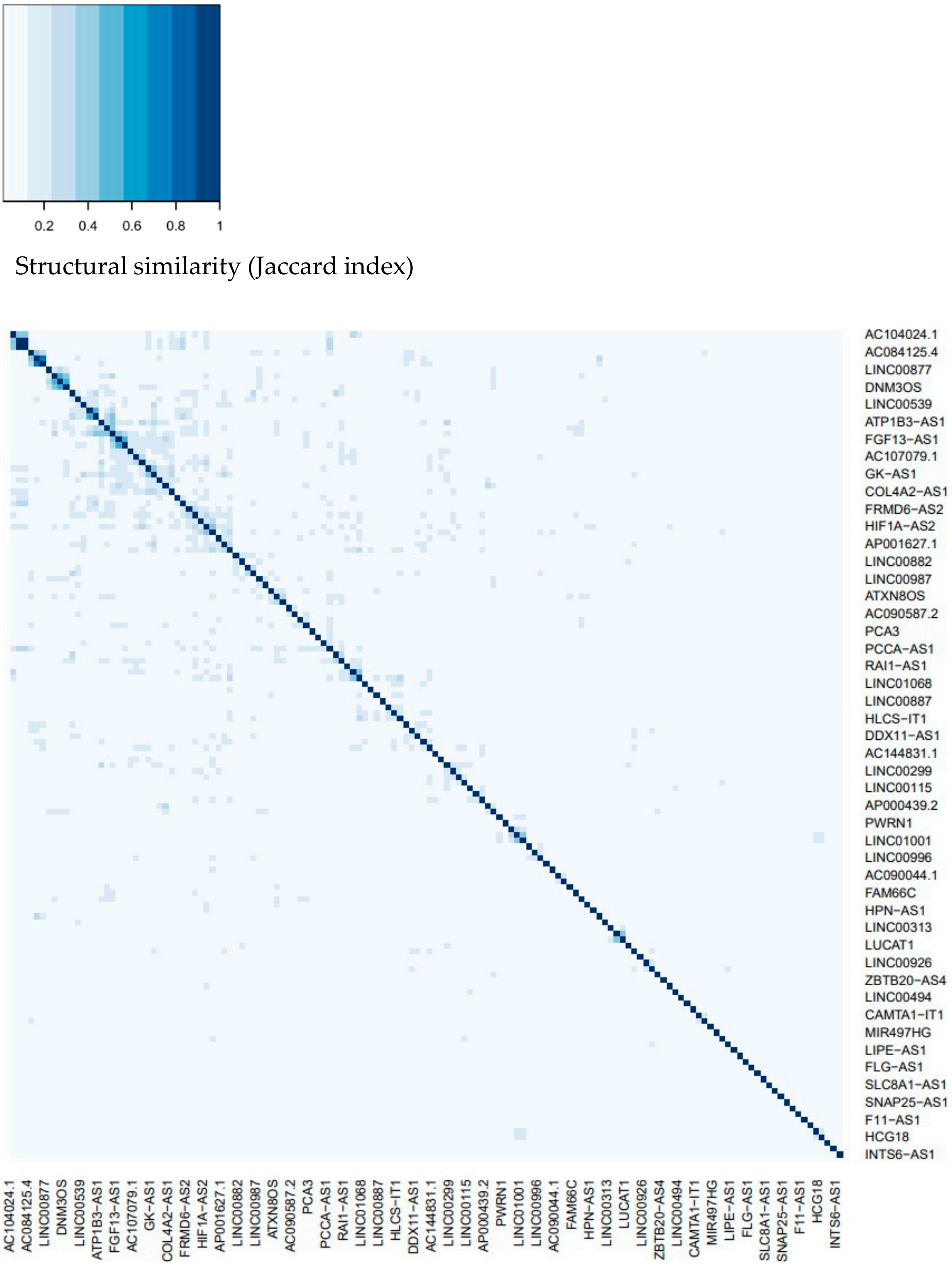

Figure S7. Structural equivalence of lncRNAs and their target mRNAs. Similarity plot of lncRNAs clustered by mRNA target similarity. Darker colors represent higher similarity among the target mRNAs.

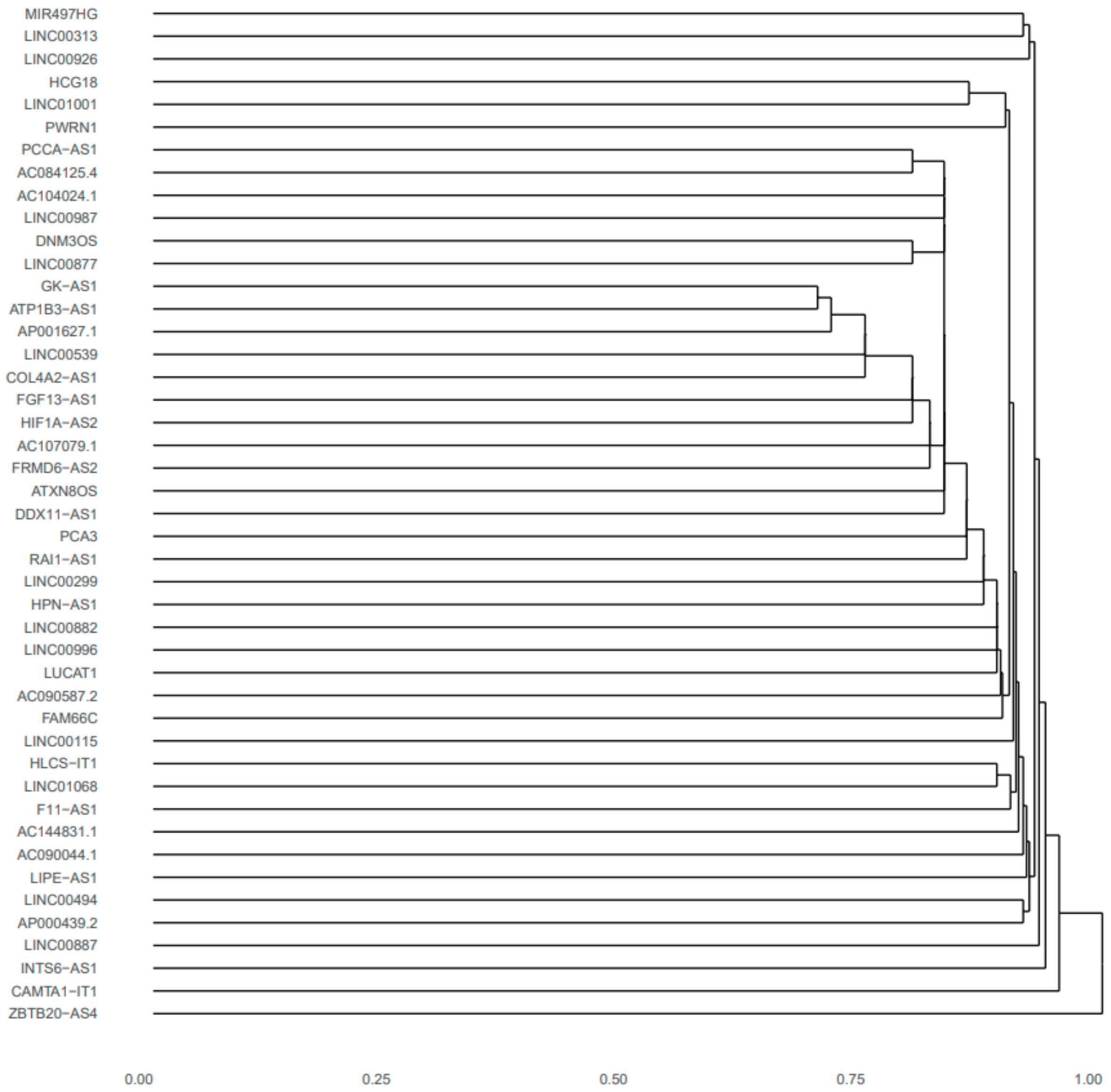

Figure S8. Structural equivalence of lncRNAs. Dendrogram plot of lncRNAs based on the number of shared mRNA targets. The x-axis represents the Jaccard index scores.

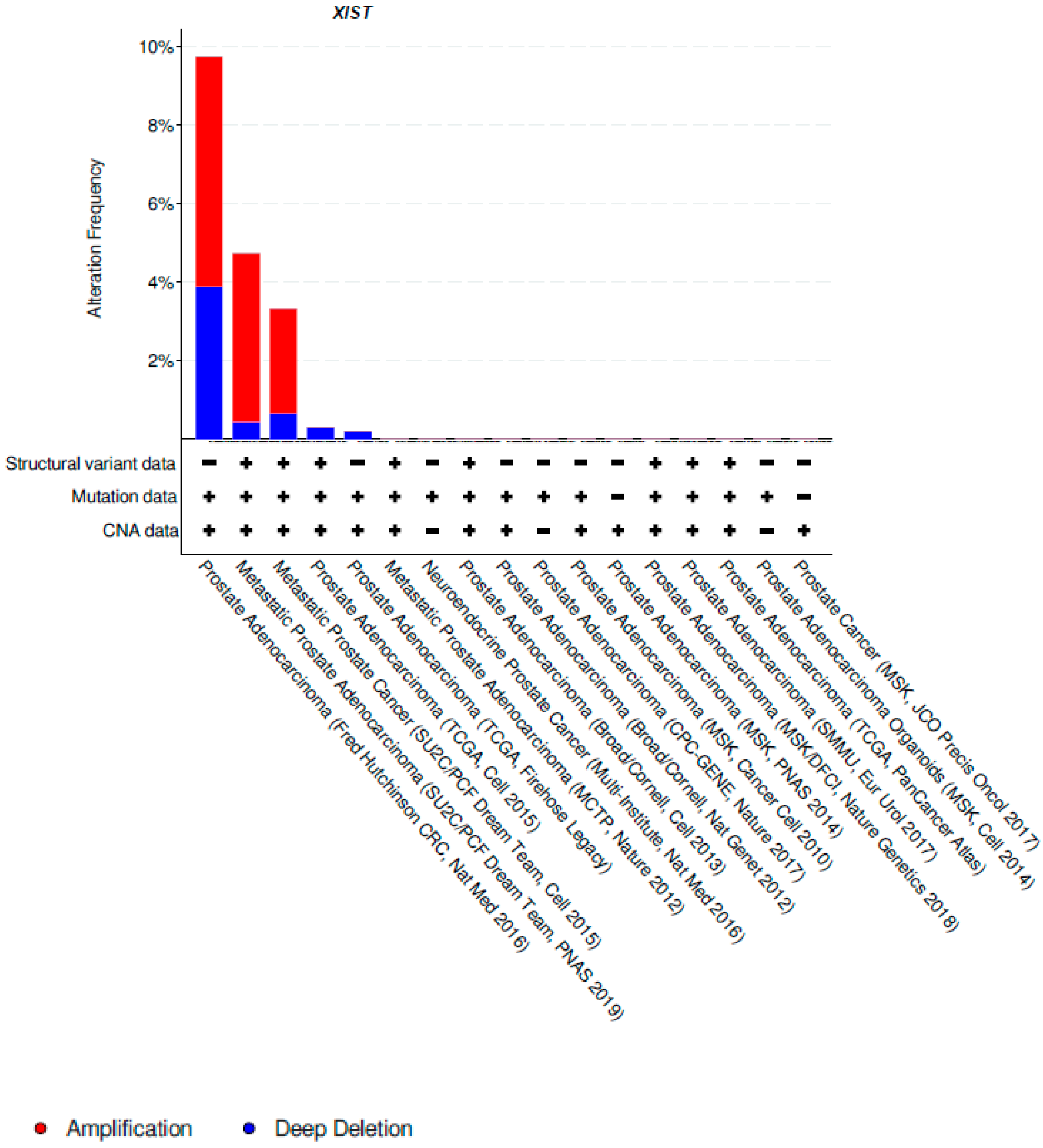

Figure S9. Alteration frequency of *XIST* across various prostate cancer datasets. The bar chart shows the percentage of samples with alterations, categorized as structural variant data, mutation data, and copy number alteration (CNA) data. Bars in RED represent upregulated alterations, while bars in BLUE represent downregulated alterations. The matrix below the chart indicates the availability of data types (+ indicates data availability, - indicates data absence) for each dataset. The datasets are labeled along the x-axis, representing studies from various sources, including TCGA and MSK, among others.

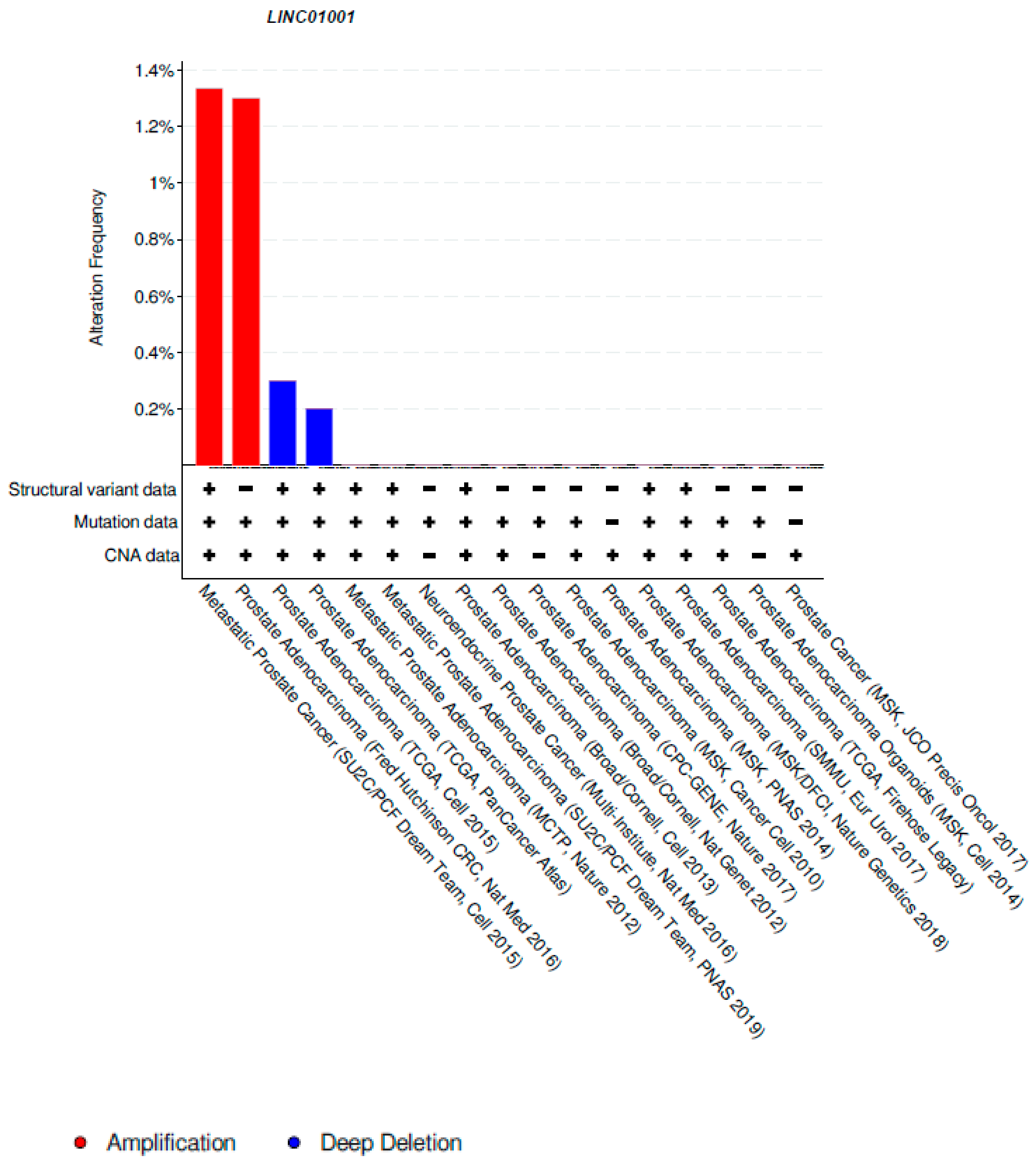

Figure S10. Alteration frequency of *LINC01001* across various prostate cancer datasets. The bar chart shows the percentage of samples with alterations, categorized as structural variant data, mutation data, and copy number alteration (CNA) data. Bars in RED represent upregulated alterations, while bars in BLUE represent downregulated alterations. The matrix below the chart indicates the availability of data types (+ indicates data availability, - indicates data absence) for each dataset. The datasets are labeled along the x-axis, representing studies from various sources, including TCGA and MSK, among others.

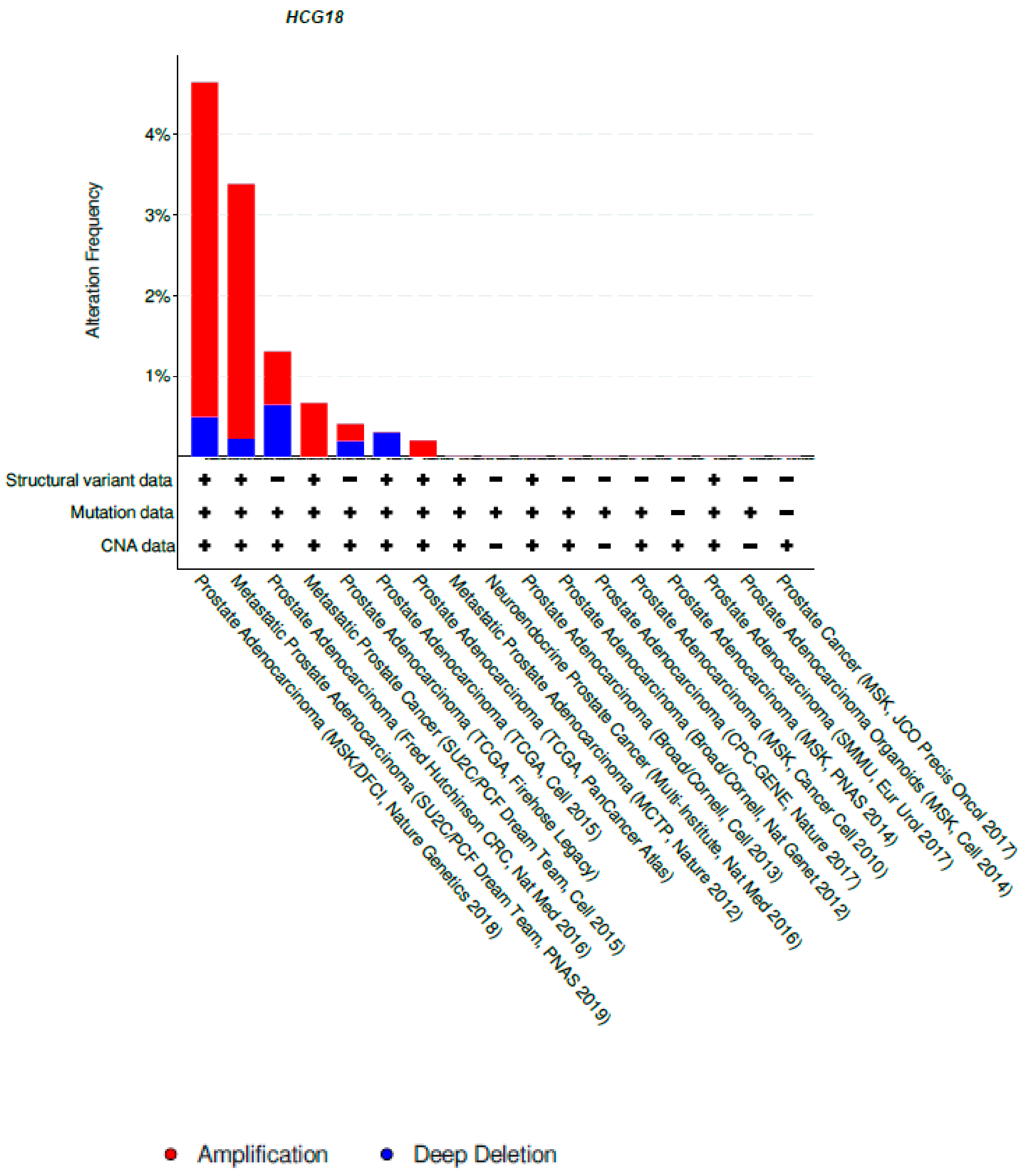

Figure S11. Alteration frequency of *HCG18* across various prostate cancer datasets. The bar chart shows the percentage of samples with alterations, categorized as structural variant data, mutation data, and copy number alteration (CNA) data. Bars in RED represent upregulated alterations, while bars in BLUE represent downregulated alterations. The matrix below the chart indicates the availability of data types (+ indicates data availability, - indicates data absence) for each dataset. The datasets are labeled along the x-axis, representing studies from various sources, including TCGA and MSK, among others.

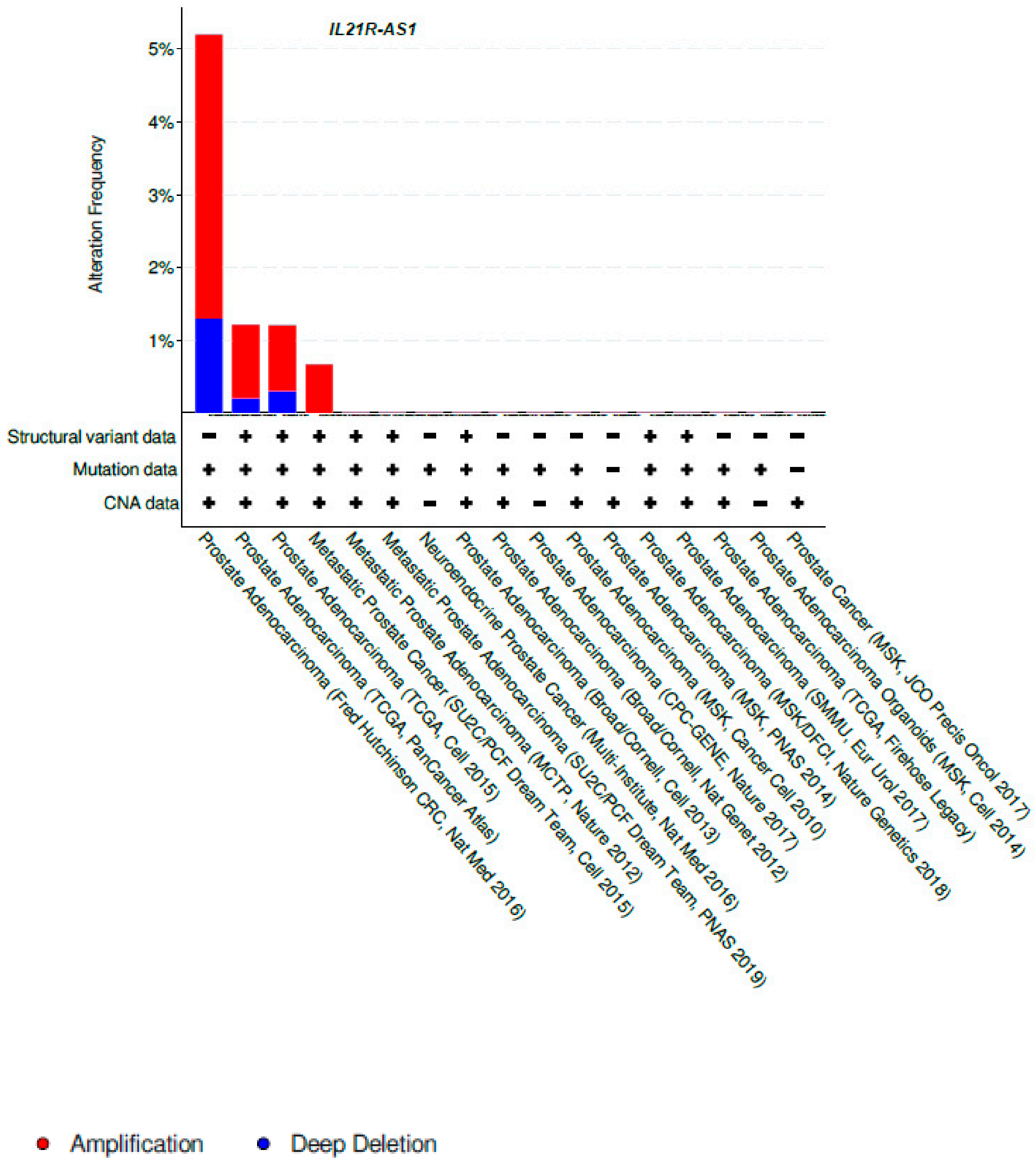

Figure S12. Alteration frequency of *IL21R-AS1* across various prostate cancer datasets. The bar chart shows the percentage of samples with alterations, categorized as structural variant data, mutation data, and copy number alteration (CNA) data. Bars in RED represent upregulated alterations, while bars in BLUE represent downregulated alterations. The matrix below the chart indicates the availability of data types (+ indicates data availability, - indicates data absence) for each dataset. The datasets are labeled along the x-axis, representing studies from various sources, including TCGA and MSK, among others.

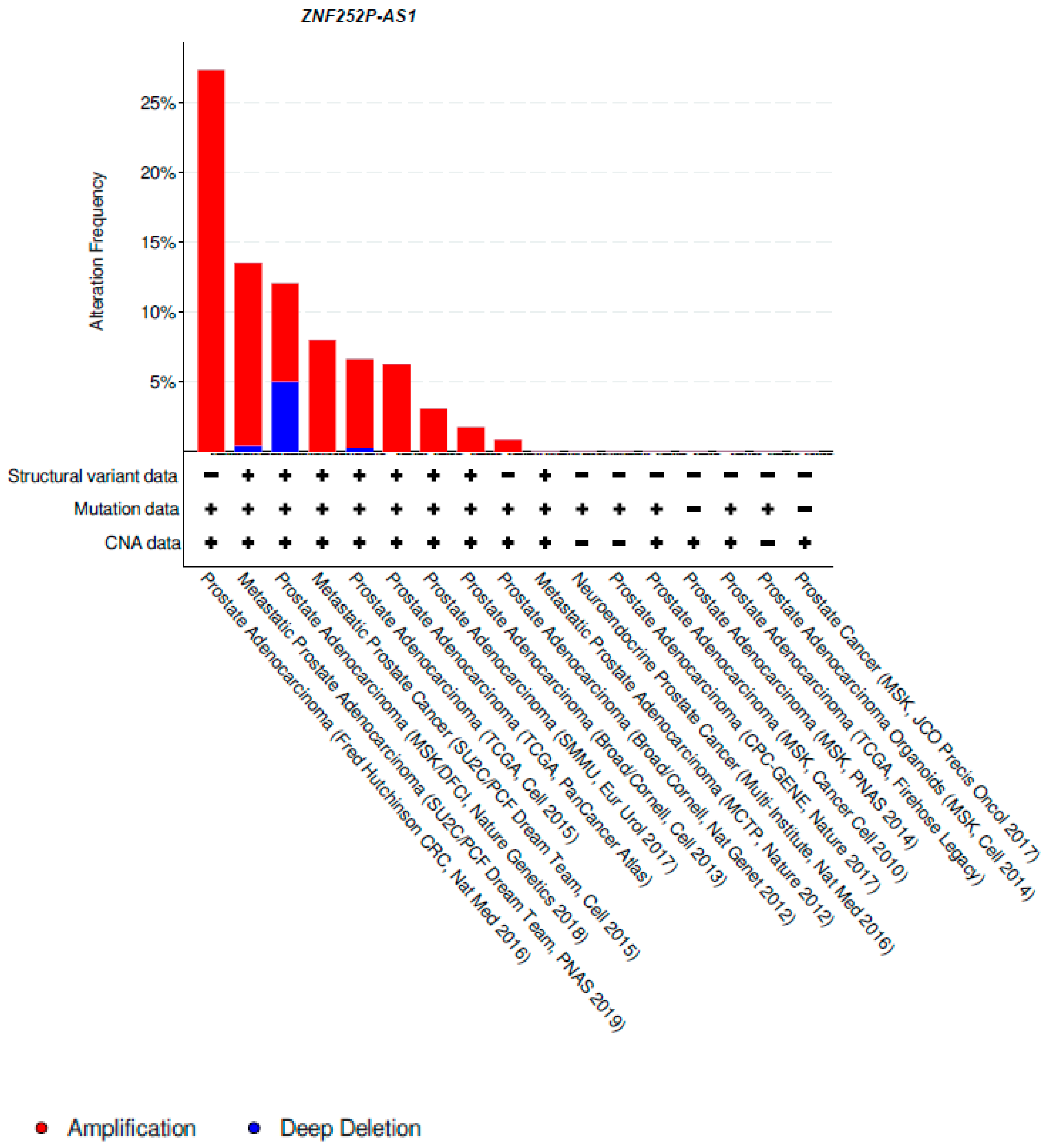

Figure S13. Alteration frequency of *ZN252P-AS1* across various prostate cancer datasets. The bar chart shows the percentage of samples with alterations, categorized as structural variant data, mutation data, and copy number alteration (CNA) data. Bars in RED represent upregulated alterations, while bars in BLUE represent downregulated alterations. The matrix below the chart indicates the availability of data types (+ indicates data availability, - indicates data absence) for each dataset. The datasets are labeled along the x-axis, representing studies from various sources, including TCGA and MSK, among others.

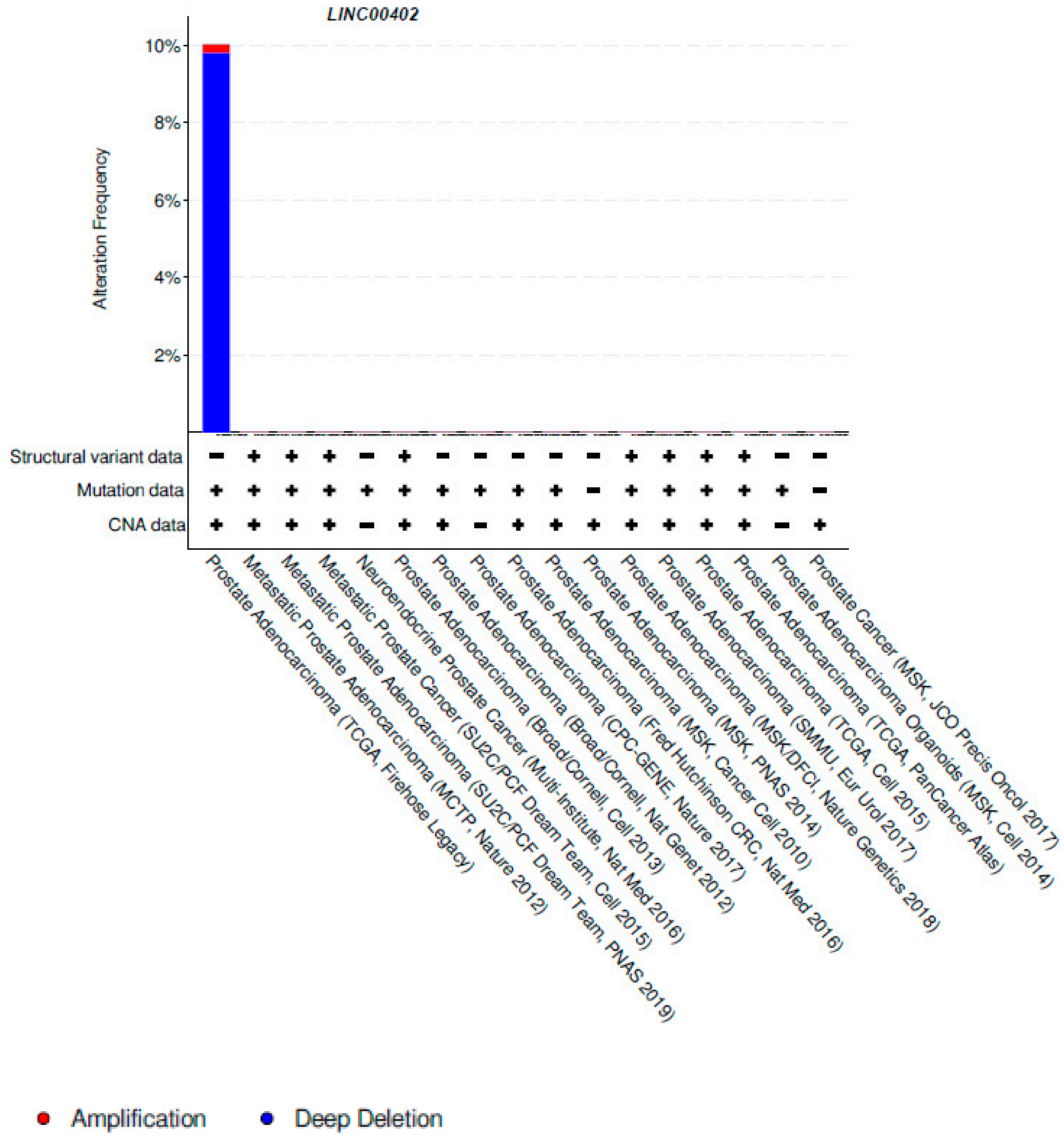

Figure S14. Alteration frequency of *LINC00402* across various prostate cancer datasets. The bar chart shows the percentage of samples with alterations, categorized as structural variant data, mutation data, and copy number alteration (CNA) data. Bars in RED represent upregulated alterations, while bars in BLUE represent downregulated alterations. The matrix below the chart indicates the availability of data types (+ indicates data availability, - indicates data absence) for each dataset. The datasets are labeled along the x-axis, representing studies from various sources, including TCGA and MSK, among others.

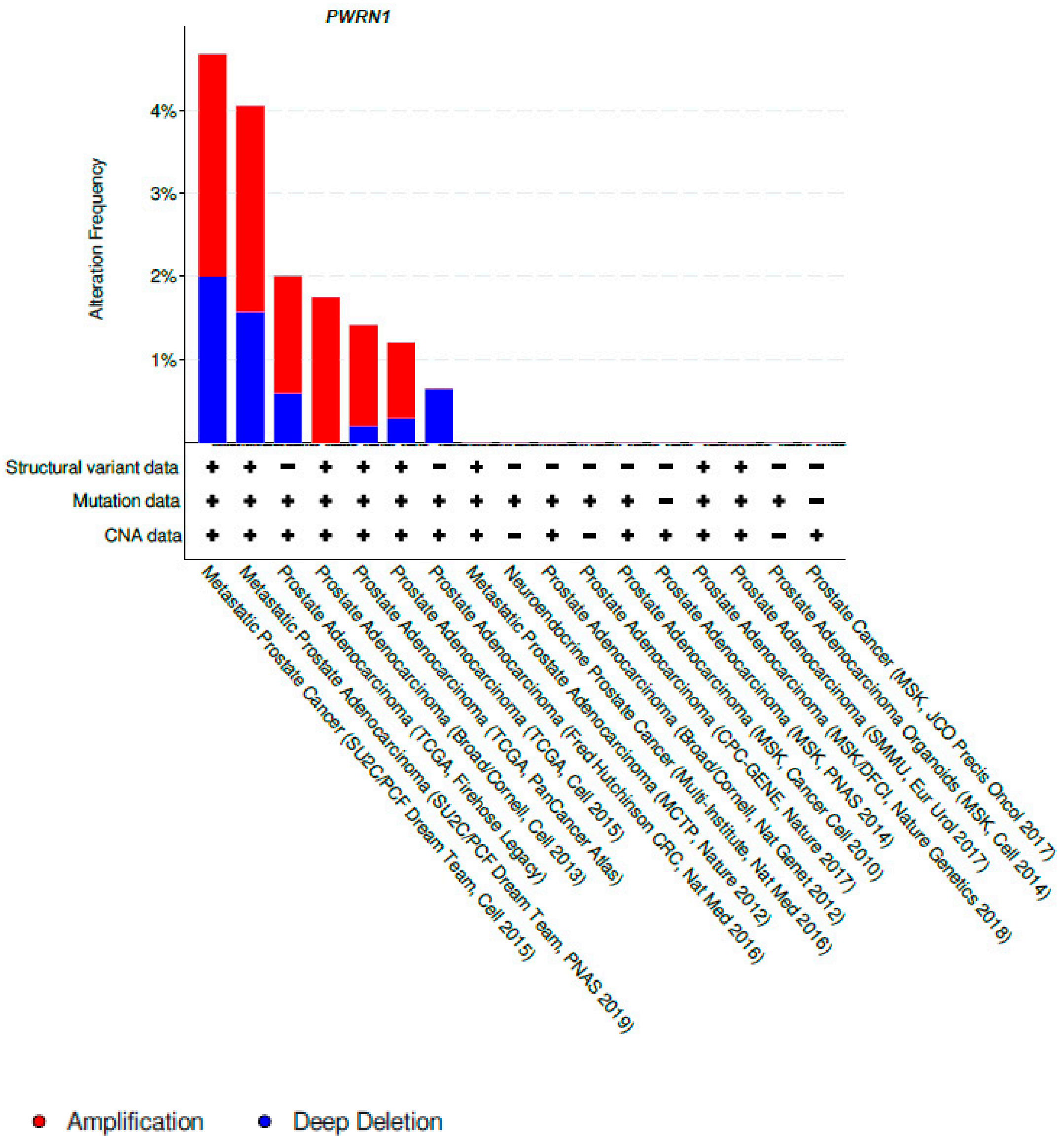

Figure S15. Alteration frequency of *PWRN1* across various prostate cancer datasets. The bar chart shows the percentage of samples with alterations, categorized as structural variant data, mutation data, and copy number alteration (CNA) data. Bars in RED represent upregulated alterations, while bars in BLUE represent downregulated alterations. The matrix below the chart indicates the availability of data types (+ indicates data availability, - indicates data absence) for each dataset. The datasets are labeled along the x-axis, representing studies from various sources, including TCGA and MSK, among others.

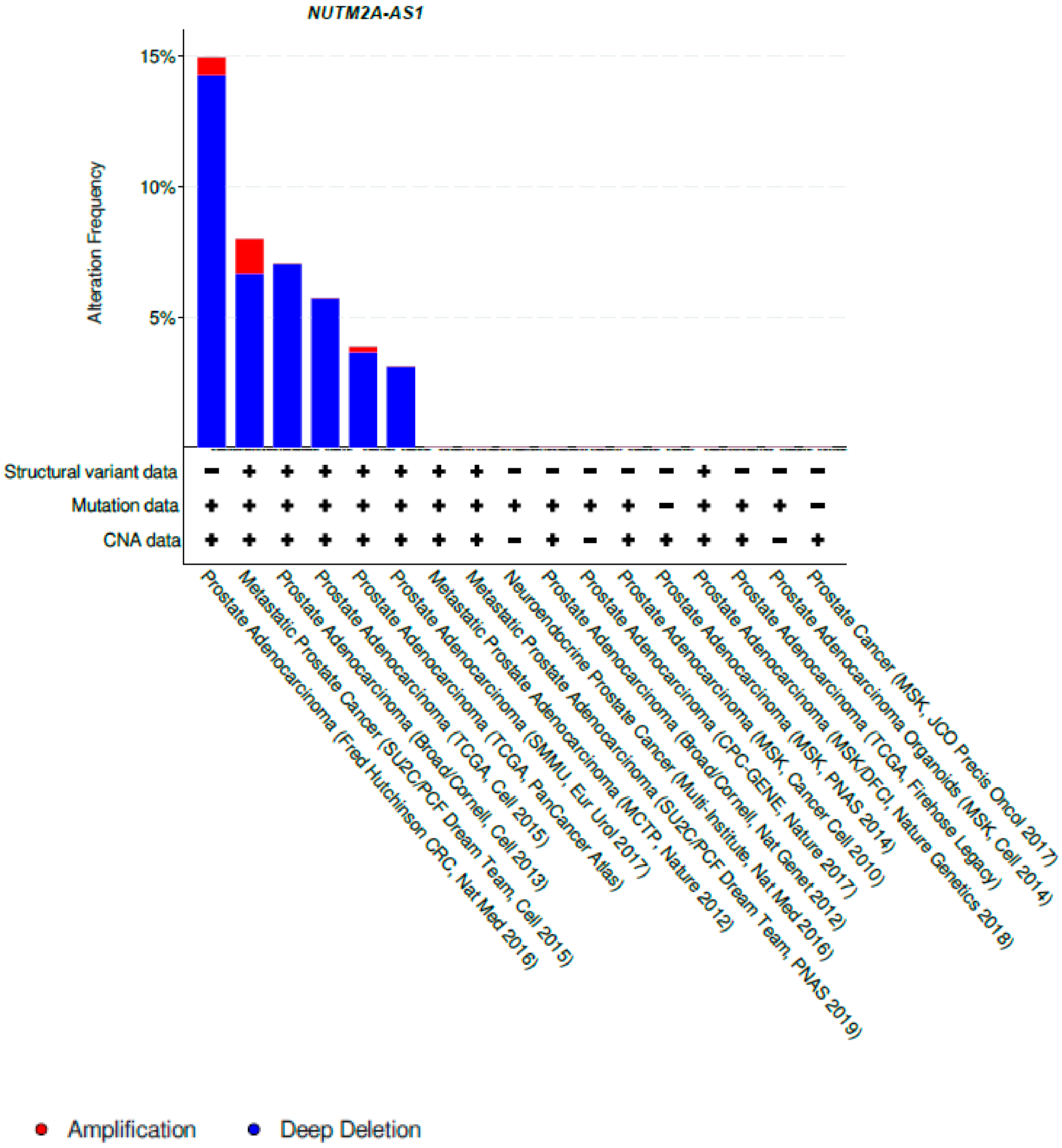

Figure S16. Alteration frequency of *NUTM2A-AS1* across various prostate cancer datasets. The bar chart shows the percentage of samples with alterations, categorized as structural variant data, mutation data, and copy number alteration (CNA) data. Bars in RED represent upregulated alterations, while bars in BLUE represent downregulated alterations. The matrix below the chart indicates the availability of data types (+ indicates data availability, - indicates data absence) for each dataset. The datasets are labeled along the x-axis, representing studies from various sources, including TCGA and MSK, among others.

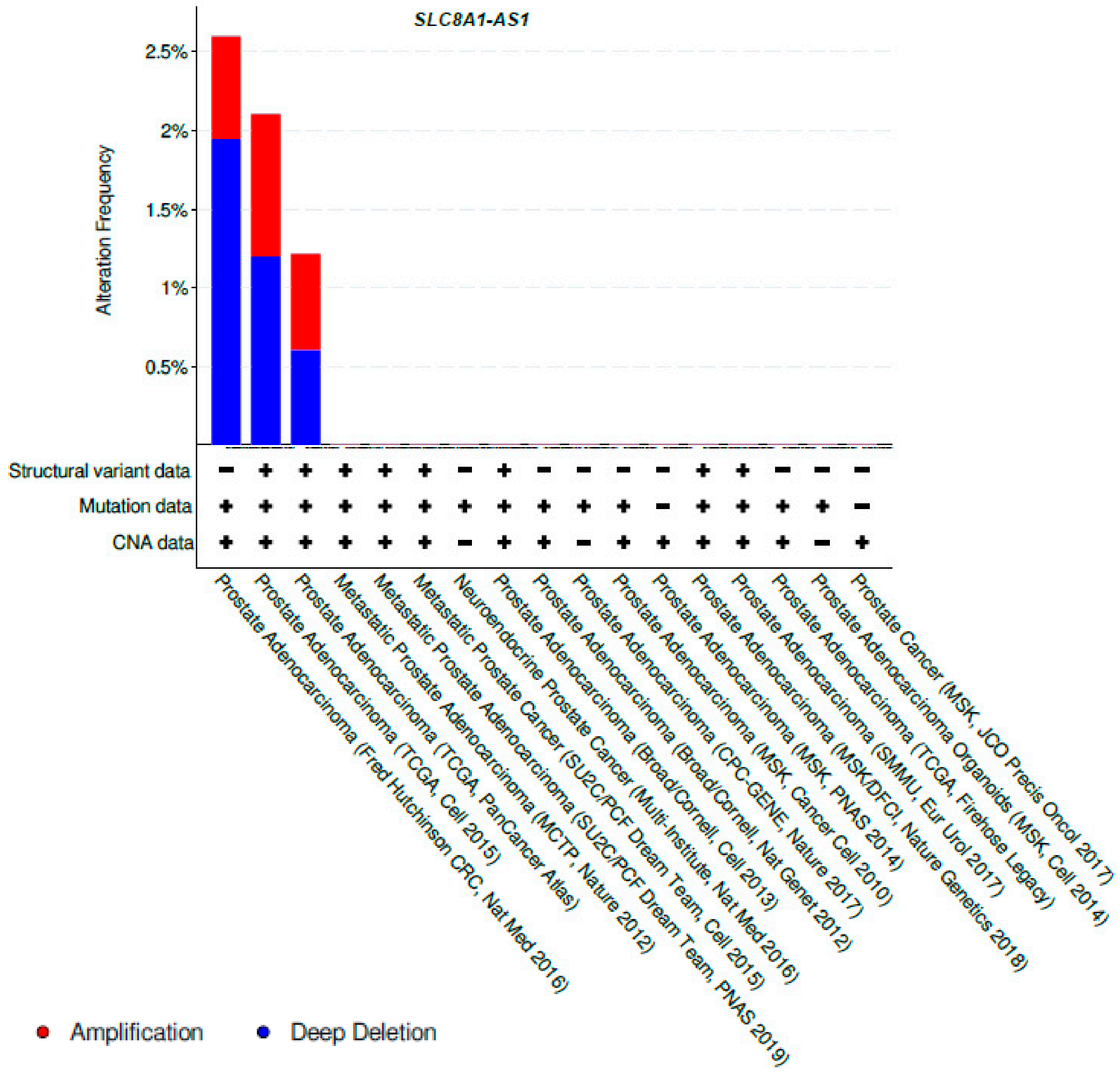

Figure S17. Alteration frequency of *SLC8A1-AS1* across various prostate cancer datasets. The bar chart shows the percentage of samples with alterations, categorized as structural variant data, mutation data, and copy number alteration (CNA) data. Bars in RED represent upregulated alterations, while bars in BLUE represent downregulated alterations. The matrix below the chart indicates the availability of data types (+ indicates data availability, - indicates data absence) for each dataset. The datasets are labeled along the x-axis, representing studies from various sources, including TCGA and MSK, among others.

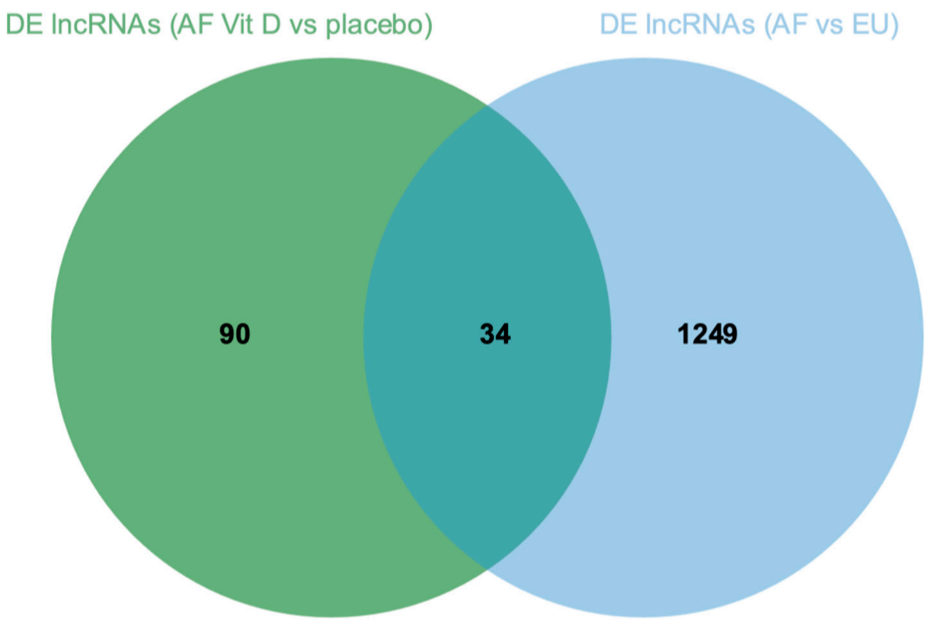

Figure S18. Area-proportional Venn diagram highlighting the overlap of the DE lncRNAs by race and vitamin D3 supplementation ($q \leq 0.4$).

Supplemental Tables (ST)

Table S1: Patient's Clinical Characteristics

Table S2 CbioPortal PC datasets queried. PC datasets queried within CbioPortal for the top 11 ranking lncRNAs identified in AF men.

Table S3: Significantly differentially expressed transcripts - AF vs EU (≤ 0.1)

Table S4: Significantly differentially expressed lncRNAs - AF vs EU (≤ 0.1)

Table S5: Significantly differentially expressed lncRNAs identified to have mRNA targets - AF vs EU (≤ 0.1)

Table S6: Significantly differentially expressed lncRNAs identified to have mRNA targets - AF vs EU (≤ 0.1) *additional info

Table S7: Over representation analysis results (lncRNA-mRNA targets - AF vs EU (≤0.1)

Table S8: lncMapper results (top ranking lncRNAs with the most mRNA interactions)

Table S9: Top ranking lncRNAs and target mRNA results merged with iPathwayGuide Immune Response gene lists

Table S10: CatRapid results - top ranking lncRNAs and their predicted protein interactions

Table S11: Significantly differentially transcripts between AF men supplemented with Vitamin D and AF men who received a placebo ($q \leq 0.4$)

Table S12: Significantly differentially expressed lncRNAs- AF vitamin D vs AF placebo (≤0.4)

Table S13: 34 Overlapping lncRNAs between AF vs EU & AF vitamin D supplemented vs placebo

Table S14: mRNA targets of the differentially expressed lncRNAs (AF vitamin D supplemented vs placebo)

References

1. Yuan, J.; Kensler, K.H.; Hu, Z.; Zhang, Y.; Zhang, T.; Jiang, J.; Xu, M.; Pan, Y.; Long, M.; Montone, K.T., et al. Integrative comparison of the genomic and transcriptomic landscape between prostate cancer patients of predominantly African or European genetic ancestry. *PLoS Genet* **2020**, *16*, e1008641, doi:10.1371/journal.pgen.1008641.
2. Rahmatpanah, F.; Robles, G.; Lilly, M.; Keane, T.; Kumar, V.; Mercola, D.; Randhawa, P.; McClelland, M. RNA expression differences in prostate tumors and tumor-adjacent stroma between Black and White Americans. *Oncotarget* **2021**, *12*, 1457-1469, doi:10.18632/oncotarget.28024.
3. Rayford, W.; Beksac, A.T.; Alger, J.; Alshalalfa, M.; Ahmed, M.; Khan, I.; Falagario, U.G.; Liu, Y.; Davicioni, E.; Spratt, D.E., et al. Comparative analysis of 1152 African-American and European-American men with prostate cancer identifies distinct genomic and immunological differences. *Commun Biol* **2021**, *4*, 670, doi:10.1038/s42003-021-02140-y.
4. Agostini, F.; Zanzoni, A.; Klus, P.; Marchese, D.; Cirillo, D.; Tartaglia, G.G. catRAPID omics: a web server for large-scale prediction of protein-RNA interactions. *Bioinformatics* **2013**, *29*, 2928-2930, doi:10.1093/bioinformatics/btt495.
5. Khachane, A.N.; Harrison, P.M. Mining mammalian transcript data for functional long non-coding RNAs. *PLoS One* **2010**, *5*, e10316, doi:10.1371/journal.pone.0010316.
6. Prostate Adenocarcinoma (TCGA, Firehose Legacy).
7. Gao, J.; Aksoy, B.A.; Dogrusoz, U.; Dresdner, G.; Gross, B.; Sumer, S.O.; Sun, Y.; Jacobsen, A.; Sinha, R.; Larsson, E., et al. Integrative analysis of complex cancer genomics and clinical profiles using the cBioPortal. *Sci Signal* **2013**, *6*, pl1, doi:10.1126/scisignal.2004088.
8. da Silveira, W.A.; Renaud, L.; Hazard, E.S.; Hardiman, G. miRNA and lncRNA Expression Networks Modulate Cell Cycle and DNA Repair Inhibition in Senescent Prostate Cells. *Genes (Basel)* **2022**, *13*, doi:10.3390/genes13020208.
