# Supplementary material for: Unveiling Racial Disparities in Localized Prostate Cancer: A Systems-Level Exploration of the lncRNA Landscape
Source: Genes (Basel). 2025 Feb 17;16(2):229. doi: 10.3390/genes16020229 (PMC11855151; doi:10.3390/genes16020229)
Supplement: Supplementary file 1 [file genes-16-00229-s001.zip › Supplemental_Table_S2.pdf]

CbioPortal Datasets queried
Prostate Adenocarcinoma (MSK/DFCI, Nature Genetics 2018)[1]
Metastatic Prostate Adenocarcinoma (SU2C/PCF Dream Team, PNAS 2019)[2]
Prostate Adenocarcinoma (Fred Hutchinson CRC, Nat Med 2016)[3]
Metastatic Prostate Cancer (SU2C/PCF Dream Team, Cell 2015)[4]
Prostate Adenocarcinoma (TCGA, Firehose Legacy)[5]
Prostate Adenocarcinoma (TCGA, Cell 2015)[6]
Prostate Adenocarcinoma (TCGA, PanCancer Atlas)[7]
Metastatic Prostate Adenocarcinoma (MCTP, Nature 2012)[8]
Neuroendocrine Prostate Cancer (Multi-Institute, Nat Med 2016)[9]
Prostate Adenocarcinoma (Broad/Cornell, Cell 2013) [10]
Prostate Adenocarcinoma (Broad/Cornell, Nat Genet 2012) [11]
Prostate Adenocarcinoma (CPC-GENE, Nature 2017) [12]
Prostate Adenocarcinoma (MSK, Cancer Cell 2010) [13]
Prostate Adenocarcinoma (MSK, PNAS 2014) [14]
Prostate Adenocarcinoma (SMMU, Eur Urol 2017) [15]
Prostate Adenocarcinoma Organoids (MSK, Cell 2014) [16]
Prostate Cancer (MSK, JCO Precis Oncol 2017) [17]

Supplemental Table 2. CbioPortal PC datasets queried. PC datasets queried within CbioPortal for the top 11 ranking lncRNAs identified in AF men. Citations are provided and numbered 1-17.

References

1. Armenia, J.; Wankowicz, S.A.; Liu, D.; Gao, J.; Kundra, R.; Reznik, E.; Chatila, W.K.; Chakravarty, D.; Han, G.C.; Coleman, I. The long tail of oncogenic drivers in prostate cancer. *Nature genetics* **2018**, *50*, 645-651.
2. Abida, W.; Cyrta, J.; Heller, G.; Prandi, D.; Armenia, J.; Coleman, I.; Cieslik, M.; Benelli, M.; Robinson, D.; Van Allen, E.M. Genomic correlates of clinical outcome in advanced prostate cancer. *Proceedings of the National Academy of Sciences* **2019**, *116*, 11428-11436.
3. Kumar, A.; Coleman, I.; Morrissey, C.; Zhang, X.; True, L.D.; Gulati, R.; Etzioni, R.; Bolouri, H.; Montgomery, B.; White, T. Substantial interindividual and limited intraindividual

genomic diversity among tumors from men with metastatic prostate cancer. *Nature medicine* **2016**, *22*, 369-378.
4. Robinson, D.; Van Allen, E.M.; Wu, Y.-M.; Schultz, N.; Lonigro, R.J.; Mosquera, J.-M.; Montgomery, B.; Taplin, M.-E.; Pritchard, C.C.; Attard, G. Integrative clinical genomics of advanced prostate cancer. *Cell* **2015**, *161*, 1215-1228.
5. Prostate Adenocarcinoma (TCGA, Firehose Legacy).
6. Cancer Genome Atlas Research, N. The Molecular Taxonomy of Primary Prostate Cancer. *Cell* **2015**, *163*, 1011-1025, doi:10.1016/j.cell.2015.10.025.
7. Hoadley, K.A.; Yau, C.; Hinoue, T.; Wolf, D.M.; Lazar, A.J.; Drill, E.; Shen, R.; Taylor, A.M.; Cherniack, A.D.; Thorsson, V. Cell-of-origin patterns dominate the molecular classification of 10,000 tumors from 33 types of cancer. *Cell* **2018**, *173*, 291-304. e296.
8. Grasso, C.S.; Wu, Y.-M.; Robinson, D.R.; Cao, X.; Dhanasekaran, S.M.; Khan, A.P.; Quist, M.J.; Jing, X.; Lonigro, R.J.; Brenner, J.C. The mutational landscape of lethal castration-resistant prostate cancer. *Nature* **2012**, *487*, 239-243.
9. Beltran, H.; Prandi, D.; Mosquera, J.M.; Benelli, M.; Puca, L.; Cyrta, J.; Marotz, C.; Giannopoulou, E.; Chakravarthi, B.V.; Varambally, S., et al. Divergent clonal evolution of castration-resistant neuroendocrine prostate cancer. *Nat Med* **2016**, *22*, 298-305, doi:10.1038/nm.4045.
10. Baca, S.C.; Prandi, D.; Lawrence, M.S.; Mosquera, J.M.; Romanel, A.; Drier, Y.; Park, K.; Kitabayashi, N.; MacDonald, T.Y.; Ghandi, M. Punctuated evolution of prostate cancer genomes. *Cell* **2013**, *153*, 666-677.
11. Barbieri, C.E.; Baca, S.C.; Lawrence, M.S.; Demichelis, F.; Blattner, M.; Theurillat, J.-P.; White, T.A.; Stojanov, P.; Van Allen, E.; Stransky, N. Exome sequencing identifies recurrent SPOP, FOXA1 and MED12 mutations in prostate cancer. *Nature genetics* **2012**, *44*, 685-689.
12. Fraser, M.; Sabelnykova, V.Y.; Yamaguchi, T.N.; Heisler, L.E.; Livingstone, J.; Huang, V.; Shiah, Y.-J.; Yousif, F.; Lin, X.; Masella, A.P. Genomic hallmarks of localized, non-indolent prostate cancer. *Nature* **2017**, *541*, 359-364.
13. Taylor, B.S.; Schultz, N.; Hieronymus, H.; Gopalan, A.; Xiao, Y.; Carver, B.S.; Arora, V.K.; Kaushik, P.; Cerami, E.; Reva, B. Integrative genomic profiling of human prostate cancer. *Cancer cell* **2010**, *18*, 11-22.
14. Hieronymus, H.; Schultz, N.; Gopalan, A.; Carver, B.S.; Chang, M.T.; Xiao, Y.; Heguy, A.; Huberman, K.; Bernstein, M.; Assel, M. Copy number alteration burden predicts prostate cancer relapse. *Proceedings of the National Academy of Sciences* **2014**, *111*, 11139-11144.
15. Chua, M.L.K.; Lo, W.; Pintilie, M.; Murgic, J.; Lalonde, E.; Bhandari, V.; Mahamud, O.; Gopalan, A.; Kweldam, C.F.; van Leenders, G., et al. A Prostate Cancer "Nimbosus": Genomic Instability and SChLAP1 Dysregulation Underpin Aggression of Intraductal and Cribriform Subpathologies. *Eur Urol* **2017**, *72*, 665-674, doi:10.1016/j.eururo.2017.04.034.
16. Gao, D.; Vela, I.; Sboner, A.; Iaquinta, P.J.; Karthaus, W.R.; Gopalan, A.; Dowling, C.; Wanjala, J.N.; Undvall, E.A.; Arora, V.K. Organoid cultures derived from patients with advanced prostate cancer. *Cell* **2014**, *159*, 176-187.
17. Abida, W.; Armenia, J.; Gopalan, A.; Brennan, R.; Walsh, M.; Barron, D.; Danila, D.; Rathkopf, D.; Morris, M.; Slovin, S. Prospective genomic profiling of prostate cancer across disease states reveals germline and somatic alterations that may affect clinical decision making. *JCO precision oncology* **2017**, *1*, 1-16.
